# A Rapid Drug Resistance Genotyping Workflow for Mycobacterium tuberculosis, Using Targeted Isothermal Amplification and Nanopore Sequencing

**DOI:** 10.1128/Spectrum.00610-21

**Published:** 2021-11-24

**Authors:** Harriet D. Gliddon, Dan Frampton, Vanisha Munsamy, Jude Heaney, Thomas Pataillot-Meakin, Eleni Nastouli, Alexander S. Pym, Adrie J. C. Steyn, Deenan Pillay, Rachel A. McKendry

**Affiliations:** a London Centre for Nanotechnology, Faculty of Mathematics and Physical Sciences, University College London, London, United Kingdom; b National Public Health Speciality Training Programme, South West, United Kingdom; c Division of Infection and Immunity, University College London, London, United Kingdom; d Africa Health Research Institute, Nelson R Mandela School of Medicine, University of KwaZulu-Natal, Durban, South Africa; e Department of Virology, University College London Hospitals NHS Foundation Trust, London, United Kingdom; f Division of Medicine, University College London, London, United Kingdom; g Department of Microbiology, University of Alabama at Birmingham, Birmingham, Alabama, USA; Quest Diagnostics Nichols Institute

**Keywords:** next-generation sequencing, isothermal amplification, nanopore sequencing, tuberculosis, drug resistance

## Abstract

Phenotypic drug susceptibility testing (DST) for tuberculosis (TB) requires weeks to yield results. Although molecular tests rapidly detect drug resistance-associated mutations (DRMs), they are not scalable to cover the full genome and the many DRMs that can predict resistance. Whole-genome sequencing (WGS) methods are scalable, but if conducted directly on sputum, typically require a target enrichment step, such as nucleic acid amplification. We developed a targeted isothermal amplification-nanopore sequencing workflow for rapid prediction of drug resistance of TB isolates. We used recombinase polymerase amplification (RPA) to perform targeted isothermal amplification (37°C for 90 min) of three regions within the Mycobacterium tuberculosis genome, followed by nanopore sequencing on the MinION. We tested 29 mycobacterial genomic DNA extracts from patients with drug-resistant (DR) TB and compared our results to those of WGS by Illumina and phenotypic DST to evaluate the accuracy of prediction of resistance to rifampin and isoniazid. Amplification by RPA showed fidelity equivalent to that of high-fidelity PCR (100% concordance). Nanopore sequencing generated DRM predictions identical to those of WGS, with considerably faster sequencing run times of minutes rather than days. The sensitivity and specificity of rifampin resistance prediction for our workflow were 96.3% (95% confidence interval [CI], 81.0 to 99.9%) and 100.0% (95% CI, 15.8 to 100.0%), respectively. For isoniazid resistance prediction, the sensitivity and specificity were 100.0% (95% CI, 86.3 to 100.0%) and 100.0% (95% CI, 39.8 to 100.0%), respectively. The workflow consumable costs per sample are less than £100. Our rapid and low-cost drug resistance genotyping workflow provides accurate prediction of rifampin and isoniazid resistance, making it appropriate for use in resource-limited settings.

**IMPORTANCE** Current methods for diagnosing drug-resistant tuberculosis are time consuming, resulting in delays in patients receiving treatment and in transmission onwards. They also require a high level of laboratory infrastructure, which is often only available at centralized facilities, resulting in further delays to diagnosis and additional barriers to deployment in resource-limited settings. This article describes a new workflow that can diagnose drug-resistant TB in a shorter time, with less equipment, and for a lower price than current methods. The amount of TB DNA is first increased without the need for bulky and costly thermocycling equipment. The DNA is then read using a portable sequencer called a MinION, which indicates whether there are tell-tale changes in the DNA that indicate whether the TB strain is drug resistant. Our workflow could play an important role in the future in the fight against the public health challenge that is TB drug resistance.

## INTRODUCTION

Globally, tuberculosis (TB) is the leading cause of death due to infection (1) and antimicrobial resistance (AMR) (2). Isoniazid (INH) and rifampin (RIF) are cornerstones of TB treatment, and resistance to these two antibiotics is classed as multidrug-resistant (MDR) TB. Of the 558,000 estimated RIF-resistant (RR) and MDR TB cases incident in 2018, only 29% (160,684) were detected (1). Antibiotic resistance in Mycobacterium tuberculosis is typically caused by spontaneous chromosomal mutations. These drug-resistance-associated mutations (DRMs) are increasingly well characterized, and the relationship between genotype and phenotype increasingly well understood and catalogued (3, 4). More than 95% of RIF DRMs are associated with the *rpoB* gene of M. tuberculosis (5), the majority occurring within the 81-bp RIF resistance-determining region (RRDR) of *rpoB* (6). INH resistance is more complex, with DRMs appearing in a number of genes, with approximately 64% occurring within the *katG* gene (7). The region with the second-most-frequently observed DRMs is the *inhA* promoter and gene region, with other DRMs observed in the *ahpC*-*oxyR* intergenic region (7) and *mshA* (8).

Molecular tests have transformed TB diagnosis, yielding results in a matter of hours, and also providing information on drug resistance. The GeneXpert MTB/RIF assay (Cepheid, Sunnyvale, CA, USA) has enabled faster TB diagnosis, prediction of RIF resistance, and shortened treatment delays (9). The Genotype MTBDRplus assay (Hain Lifescience GmbH, Nehren, Germany), a line probe assay (LPA), identifies DRMs for both INH and RIF. However, gene sequencing is required for comprehensive DRM detection.

TB can be detected by whole-genome sequencing (WGS) of M. tuberculosis liquid cultures (10), which is routinely used for the full characterization of all TB cases in the United Kingdom (11, 12). However, Illumina-based sequencing requires significant cost outlays, can only sequence relatively short nucleic acid fragments, and requires long run times (typically 24 to 48 h), and real-time data analysis is not possible. In contrast, technologies like nanopore sequencing have significantly lower cost outlays and run times an order of magnitude shorter. The MinION (Oxford Nanopore Technologies, Oxford, UK) is a portable device that connects to a computer and performs analysis in real time, which could further reduce delays in diagnosis (13).

Nucleic acid amplification is typically required prior to genomic sequencing of M. tuberculosis. Isothermal amplification methods are suited to deployment in resource-limited settings because, unlike PCR, they do not require thermocyclers, and they occupy a reduced footprint in the laboratory (14, 15). Recombinase polymerase amplification (RPA) is one such technique that has been used for Illumina sequencing library preparation previously and was shown to amplify with equivalent or superior fidelity compared to high-fidelity PCR polymerases (16).

Herein, we sought to develop a rapid drug resistance-genotyping workflow for M. tuberculosis, harnessing targeted isothermal amplification and nanopore sequencing to speed time to result. We developed targeted isothermal amplification assays for three MDR-associated regions of the M. tuberculosis genome and combined these with a nanopore sequencing phase for the diagnosis of MDR TB. We aimed to assess the fidelity of amplification by RPA compared to that of high-fidelity PCR and determine whether nanopore sequencing is sufficiently accurate compared to Illumina sequencing in order to confidently call single-nucleotide polymorphisms (SNPs). The overall aim of this study was to evaluate whether our workflow of targeted RPA and nanopore sequencing can accurately predict drug resistance more rapidly than existing protocols. Importantly, this is the first study to implement RPA alongside nanopore sequencing and also the first to evaluate the fidelity of RPA for a GC-rich genome.

## RESULTS

### RPA primer design and screening.

RPA amplicons are typically designed to be between 100 and 250 bp in order to facilitate rapid amplification. Standard RPA preparations are therefore unable to produce amplicons longer than 1,500 bp (17). Since our desired amplicons were 1,300 to 1,600 bp, we used custom RPA kits provided by TwistDx which were suited to the generation of longer amplicons.

For each of the three genome regions of interest, *rpoB*, *katG*, and *inhA*, three or four forward and reverse primers were designed for screening. All primers were between 28 and 34 bp in length and had a melting temperature of 63 to 73°C and a GC content of 45 to 73% (Table S1 in the supplemental material). The primers did not contain mutations that appeared in the isolates in four databases (TBDream [18], TBVar [19], polyTB [20], and GMTV [21]) (Table S1). Primer combinations that gave the highest yield of RPA amplicons were selected for further use after confirmation of correct amplification by Illumina sequencing (Fig. S1, S2, and S3). The final *rpoB*, *inhA*, and *katG* RPAs resulted in the generation of amplicons of 1,323, 1,547, and 1,511 bp, respectively (Table S2; Fig. S4). Our data show that the custom RPA kit, optimized for the production of longer amplification products of approximately 1.5 kb, is necessary for the generation of our amplicons (Fig. S5) and that 90 min is required to allow sufficient amplification of all three amplicons (Fig. S6).

A concentration gradient of strain H37Rv genomic DNA was used to investigate the required concentration of template DNA for each RPA (Fig. S7). We also considered the yield of DNA from sputum achieved by Votintseva et al. (13), which ranged from 0.05 to 50 ng/µl. Assuming a total volume of 10 µl, that would equate to 0.5 ng to 500 ng. We therefore used 50 ng total DNA (i.e., the midpoint of this range) for each RPA.

### Illumina sequencing compared to MinION for *rpoB* amplicon.

We first compared Illumina sequencing on a MiSeq to nanopore sequencing on the MinION for the *rpoB* RPA amplicon, mapping to the H37Rv genome, in order to design a workflow for DRM identification. For the 48-h MiSeq run, the read depth of coverage was typically in excess of 10,000× across the amplicon. Despite an ∼100-fold-shorter run time, the read depth of coverage for the 30-min nanopore run was typically between 1,000× and 10,000×. Base variation on the Illumina MiSeq platform corresponded closely to Illumina’s 0.1% error rate across the amplicon, whereas nanopore variation was heteroscedastic and more broadly spread. Approximately 15% of nanopore positions showed no sequence variation at all, while many others gave variations between 1% and 10% (Fig. S8). As similar variation was not seen at these positions within the MiSeq reads, we presume these are systematic base-calling errors. Thus, while the overall sequencing error was low, given the heteroscedastic nature of the per-site variation observed in this study, accurate quantification of variants is problematic at present. However, this is beyond the scope or requirements of this study and does not impact on the ability of nanopore sequencing to reliably call consensus sequence.

Observed sequence variation is also highly dependent on the software used to map reads to the H3Rv reference strain sequence: our analysis also showed that fewer base variations were observed when Minimap2 was used than with bwa_ont, especially in regions surrounding primer-binding sites. Given these results, we decided on a 3-h sequencing run time for our study to determine the diagnostic accuracy of drug resistance prediction using the MinION.

### Clinical samples.

We used genomic DNA extracts from clinical isolates cultured from sputum samples produced by MDR- and extensively drug-resistant (XDR) TB patients in KwaZulu Natal, South Africa, collected in a previous study in which WGS and dating analysis were performed to assess the emergence of M. tuberculosis drug resistance with time (22). Illumina WGS was performed on a HiSeq 2000 platform. The results of the WGS and phenotypic DST as published were used to evaluate the results of this study (Table S3).

### Use of archived TB strains to trial RPA and nanopore sequencing workflow.

**FIG 1:**
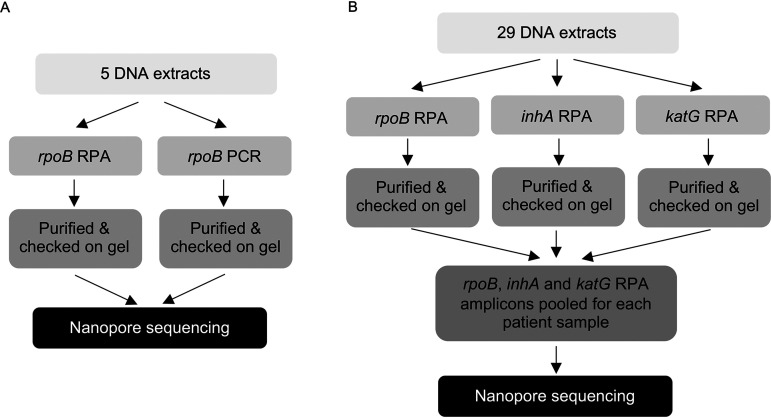
Overview of the study. (A) Investigation of fidelity of RPA polymerase compared to fidelity of PCR. DNA was amplified by either PCR or RPA prior to sequencing. (B) For the RPA/nanopore sequencing workflow, three genomic regions were amplified by RPA, purified, and then pooled prior to library preparation and nanopore sequencing.

Figure 1 outlines the two components of our study in which clinical samples were used. The first involved comparing the fidelity of the *rpoB* RPA to that of high-fidelity PCR. The second involved performing each of the *rpoB*, *inhA*, and *katG* RPAs for each sample separately before purifying and pooling the amplicons, followed by nanopore library preparation and sequencing on the MinION.

All samples were amplified (Fig. S9) and sequenced successfully using our workflow (Fig. S10). The library preparation was successful, with an average of 1.7 Mb of total reads per flow cell, 109 kb mapping to H37Rv, and 83.2% of reads barcoded. In total, four flow cells were run. All four flow cells gave similar results in quality control and started sequencing with a similar number of channels (Tables S4 and S5).

### Fidelity of RPA amplification.

A high-fidelity PCR amplification assay for *rpoB* was also designed to allow assessment of the *rpoB* RPA (Fig. S11). Five samples were chosen at random for amplification of the *rpoB* region of interest by high-fidelity PCR (mutation frequency 0.013%) and by RPA. The sequences for all five samples were entirely concordant regardless of whether PCR or RPA was used for amplification, indicating that RPA is capable of high-fidelity amplification of the GC-rich M. tuberculosis genome.

**FIG 2:**
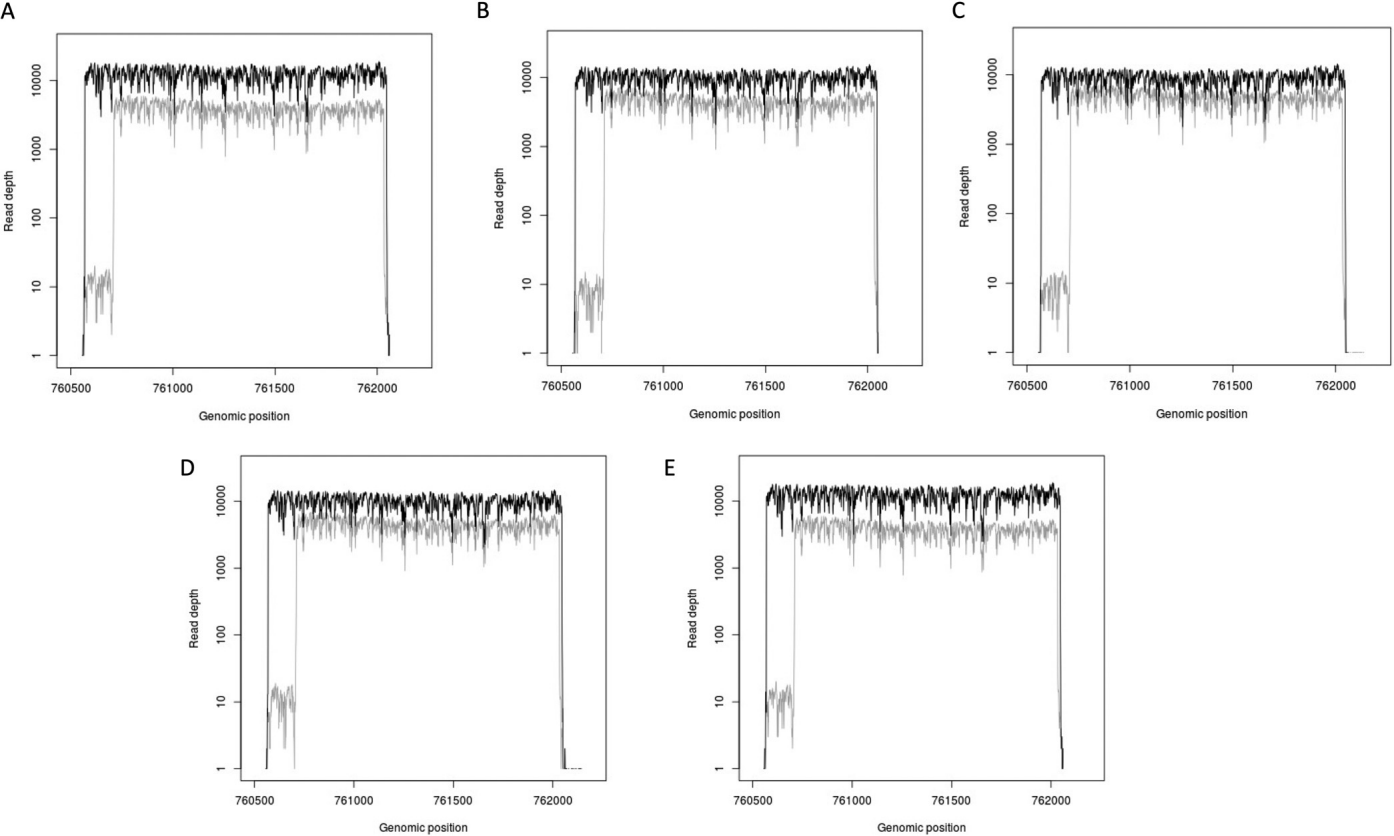
Read depths, displayed on a log scale, for PCR and RPA *rpoB* amplicons for five samples, shown by black and gray lines, respectively.

The number of reads was greater for RPA amplicons than for PCR amplicons, with mean numbers of reads of 27,019 for samples amplified by PCR and 108,033 for those amplified by RPA (Table S6). However, the depth of coverage was significantly higher for the PCR amplicons, with a median depth of coverage of 10,155, compared to 4,365 for RPA (Fig. 2), and it appears that amplification by PCR is more efficient than amplification by RPA. The coverage plots for all five PCR amplicons show a low depth of coverage for approximately 200 bp, most likely due to spurious amplification effects. The sequencing data also gave information on amplicon lengths, which were as expected (Table S7).

### Accuracy of nanopore sequencing.

We saw complete concordance between consensus amplicon sequences generated by nanopore sequencing and those previously assembled using Illumina HiSeq. The exception was a frameshift in *katG* resulting from a homopolymeric sequencing error observed in 11 of 12 samples run on a single flow cell. Given that this error occurred in this flow cell only and was not observed in samples on other flow cells, we posit that this was the consequence of a systematic base-calling error particular to the flow cell firmware used at run time that has since been updated.

The proportion of nonconsensus bases at each amplicon position was low, the median frequencies of nonconsensus bases per site across all samples being 0.25% (interquartile range [IQR], 0.24 to 0.32%), 0.30% (IQR, 0.00 to 0.32%), and 0.29% (IQR, 0.28 to 0.31%) for the *rpoB*, *katG*, and *inhA* amplicons, respectively. Importantly, this suggests that the higher rate of sequencing errors of nanopore might be overcome with relatively low depths of read coverage to accurately call mutations in consensus sequences relative to the reference.

### Nanopore sequencing results for pooled amplicons.

The overall read depth was higher on average for the *rpoB* amplicon, with the lowest coverage observed for *katG* (Tables S8 and S9). However, the *rpoB* amplicon had a higher proportion of sequences with a read depth of less than 20. The amplicon length calculated from the sequencing data was very close to the actual length of each of the amplicons.

Although the vast majority (95%) of sequenced reads do not map to the H37Rv genome, our targeted amplification appears to be highly efficient, with 89% of the nanopore reads mapping to a genomic region corresponding to one of our three RPA amplicons (Table S10). There is clearly scope for optimization; *katG* is under- and *inhA* overrepresented in terms of relative abundance of reads. However, the median depths across the amplicons are good and more than sufficient to accurately call DRMs.

### Algorithm for calling DR.

Using the DRM classification system proposed by Miotto et al. (4) we classified any sample for which a “moderate”- or “high”-confidence mutation was identified as resistant and those samples with “minimal”-confidence mutations or without mutations as susceptible (Table S11). The mutations identified by nanopore sequencing are listed in Tables 1 and 2 for RIF and INH resistance, respectively.

**TABLE 1:** RIF DRMs identified by nanopore sequencing

*rpoB* DRM	Frequency	Confidence of mutation^*a*^	Classification^*b*^
S531L	14	High	R
H526L	1	High	R
S531W	1	High	R
D516G	3	High	R
D516V	3	High	R
H526R	1	High	R
D516Y	3	Moderate	R
L533P	3	Moderate	R
L511P	1	Minimal	S
D516Del	1	Not identified	S

a According to Miotto et al. (4).

b R, RIF resistant; S, RIF susceptible.

**TABLE 2:** INH DRMs identified by nanopore sequencing

DRM	Frequency	Confidence of mutation^*a*^	Classification^*b*^
*katG* S315T	21	High	R
*inhA* c(-15)t	8	High	R
*mshA* A187V	3	Moderate	R

a According to Miotto et al. (4).

b R, INH resistant; S, INH susceptible.

The most frequently observed RIF DRM was S531L in *rpoB* (encoding a change of S to L at position 531), which occurred in 14 of the 29 strains (Table 1). Other high-confidence *rpoB* mutations that were identified included H526L, S531W, D516G, D516V, and H526R. Two moderate-confidence mutations were identified, D516Y and L533P. The presence of these high and moderate confidence mutations led to the classification of 26 samples as RIF resistant (RIF R). Each of these classifications agreed with the results of the phenotypic DST (Table S12). The L511P mutation was identified in one strain. As this was of minimal confidence, it was classified as susceptible, which was also the result given by phenotypic DST. The D516Del mutation was identified for one strain. This mutation was listed as an indeterminate mutation by Miotto and colleagues (4), possibly due to insufficient data supporting its association with resistance. Since the mutation was not classified as a high- or moderate-confidence mutation and no other mutations were identified, we classified the strain as RIF susceptible. However, this disagreed with the phenotypic DST result for this strain, which was RIF resistant.

For INH resistance, two genomic regions were assessed. These included part of the *katG* gene and the *inhA* gene, including its regulatory region. The most commonly identified DRM associated with INH resistance was the *katG* S315T mutation, which was present in 21 of the 29 strains. As this is a high-confidence mutation, all were classified as resistant. The *inhA* c(-15)t mutation, also of high confidence, was detected in eight strains. All strains with either or both of these mutations were classified as INH resistant (INH R), which was in agreement with the phenotypic DST results (Table S13).

### Accuracy of RPA/nanopore sequencing workflow for DR prediction.

We calculated the sensitivity and specificity for predictions of RIF and INH resistance. The mean values for sensitivity and specificity of RIF resistance prediction for the RPA/nanopore sequencing workflow were 96.3% (95% CI, 81.0 to 99.9%) and 100.0% (95% CI, 15.8 to 100.0%), respectively (Table 3). One strain, which harbored the D516Del mutation, was incorrectly classified as susceptible by our workflow and was therefore a false-negative detection. For two strains, no RIF DRMs were identified, and the strains were therefore correctly classified as RIF susceptible. Detailed information on all DRMs identified for each strain can be found in the supplemental material.

**TABLE 3:** Diagnostic accuracy of RIF resistance prediction by RPA/nanopore sequencing, using phenotypic DST as the reference test

Result using index method (RPA/nanopore)^*a*^	No. of strains with indicated result using reference method (DST)^*b*^				
	R	S	Total	Sensitivity (95% CI)	Specificity (95% CI)
R	26	0	26	96.30 (81.03–99.91)	100.00 (15.81–100.00)
S	1	2	3		

Total	27	2	29		

a RPA, recombinase polymerase amplification; R, RIF resistant; S, RIF susceptible.

b DST, drug susceptibility testing.

The Illumina WGS gave results identical to those of our workflow (Table S14), and the same DRMs in *rpoB* were detected in each strain. Given that the same DRMs were used to call resistance for both the Illumina WGS results and the RPA/nanopore sequencing results, the strain that possessed the D516Del mutation was again incorrectly classified as RIF susceptible, and so the sensitivity and specificity were exactly the same as those of RPA/nanopore sequencing (Table 3).

For INH resistance prediction, the sensitivity and specificity were 100.0% (95% CI, 86.3 to 100.0%) and 100.0% (95% CI, 39.8 to 100.0%), respectively (Table 4). The Illumina WGS identified the same DRMs as RPA/nanopore sequencing (Table S15). However, the Illumina results also enabled the identification of the *mshA* A187V mutation in three strains (TKK_05SA_0019, TKK_05SA_0046, and TKK_05SA_0052). This mutation is listed as a moderate-confidence mutation by Miotto et al. (4), and therefore, these strains would be classified as INH resistant according to our analysis. However, the phenotypic DST results list two of them as INH susceptible. The third strain also harbored the *katG* S315T high-confidence DRM and was correctly classified as INH resistant. A sensitivity and specificity of 100.0% (95% CI, 86.3 to 100.0%) and 50.00% (6.76% to 93.24%), respectively, were calculated for Illumina WGS. The lower specificity for Illumina WGS than for RPA/nanopore sequencing is a consequence of the *mshA* A187V mutation, which was detected by Illumina WGS but not by RPA/nanopore sequencing, since *mshA* was not a target of RPA, and our analysis classified those samples with high or moderate (as was the case for *mshA* A187V) confidence mutations as resistant.

**TABLE 4:** Diagnostic accuracy of INH resistance prediction by RPA/nanopore sequencing, using phenotypic drug susceptibility testing as the reference test

Result using index method (RPA/nanopore)^*a*^	No. of strains with indicated result using reference method (DST)^*b*^				
	R	S	Total	Sensitivity (95% CI)	Specificity (95% CI)
R	25	0	25	100.00 (86.28–100.00)	100.00 (39.76–100.00)
S	0	4	4		

Total	25	4	29		

a RPA, recombinase polymerase amplification; R, RIF resistant; S, RIF susceptible.

b DST, drug susceptibility testing.

### Time to prediction of DR using RPA/nanopore sequencing.

The results reported above were obtained from multiplexing batches of 12 samples over 3 h, corresponding to a sequencing time per sample of 15 min. Given the high read depths observed for most samples across each amplicon, we investigated whether sequencing could be performed more rapidly while still generating reliable sequences by using subsets of reads from three different samples, selected as having a high, median, and low depth of coverage relative to the read depths of all samples (Table S16). The read subsets consisted of 1, 5, 10, 25, and 50% of the total reads for each sample, corresponding to sequencing times ranging from 9 s to 7.5 min. For each subset, we compared the final consensus sequences for each amplicon with those generated from the complete set of sample reads, assuming that any differences were due to insufficient read coverage to generate an accurate consensus.

We were able to generate accurate amplicon sequences for all three samples using only 10% of the total reads, corresponding to a sequencing time of ∼90 s per sample. Where read depths were higher, we were able to use a smaller proportion of reads. There were no errors in any of the full-length amplicon sequences produced where the median read depth was 20 reads or higher (Table S17). If this were to be scaled up, it would correspond to a multiplexed batch of 12 samples requiring a run time of 18 min, the equivalent of a 96-well plate requiring just under 2.5 h to sequence. This is considerably less time than is needed to perform Illumina sequencing, which typically takes 24 to 48 h per 96-well plate run. While the relatively higher error rate of nanopore sequencing may prohibit accurate variant calling, for the purposes of generating reliable consensus sequences and calling DRMs, lower depths of coverage (corresponding to shorter run times) would appear to suffice.

### Cost of DR prediction using RPA/nanopore sequencing.

We calculated the overall consumable cost per sample of the RPA/nanopore sequencing workflow, including the MolYsis DNA extraction kit that was used by Votintseva et al. for differential lysis and extraction of mycobacterial DNA from sputum samples (13). Assuming samples were barcoded and 12 were run on each flow cell, the total consumable cost per sample was GBP98.39 (Table S18). Given the low outset costs associated with nanopore sequencing, which are generally associated with establishing the computer hardware equipment rather than the sequencing device, this is a considerably more affordable and accessible technology and workflow than others currently available.

## DISCUSSION

**FIG 3:**
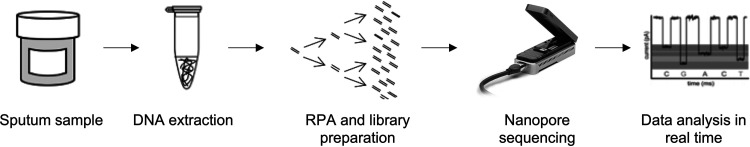
Proposed sample-to-answer workflow.

In this study, we have developed and evaluated a new rapid workflow for targeted isothermal amplification of drug resistance-associated genomic regions of M. tuberculosis followed by nanopore sequencing. We have shown there is no reduced accuracy of amplification for isothermal RPA and MinION sequencing of mycobacterial DNA compared to the accuracy of the more common but more costly and time-consuming approach of PCR amplification and Illumina sequencing. Indeed, the MinION-derived consensus sequences were identical to their HiSeq counterparts and required significantly shorter run times (2.5 h compared to 24 to 48 h) for DR prediction. Thus, our rapid and low-cost workflow is as capable of accurately identifying existing DRMs as existing sequencing approaches (Fig. 3).

Our results show that RPA generates amplicons with accuracy similar to that of high-fidelity PCR. The RPA cycle used in this work requires 90 min at 37°C. The constant temperature required for RPA negates the need for thermocyclers and a stable power supply, as required by PCR methods. The PCR cycle used for the generation of *rpoB* amplicons in this work also required an overall time of 90 min (although this is dependent on the ramp rate of a thermocycler). However, RPA has proved less prone to inhibition than PCR (23), indicating that simpler DNA extraction processes could be used prior to amplification by RPA.

We have demonstrated that nanopore sequencing is capable of generating sufficient genomic output to reliably call DRMs in ∼90 s per sample. Scaling up, this corresponds to an overall sequencing run time ∼10 to 20 times shorter than the time for running a 96-well sample plate on an Illumina MiSeq, a significant decrease in time to prediction for individual samples and multiplexed batches.

Our workflow identified resistance markers for two key anti-TB drugs, RIF and INH. However, our workflow is not limited to specific genes or genomic regions and is scalable to cover a variety of regions within the M. tuberculosis genome where other DRMs are known to occur. We have shown that combining several RPA amplicons is possible, without reducing the fidelity of subsequent sequencing on the MinION. Therefore, our workflow should be readily extended to incorporate additional genes and regions of interest, such as *oxyR-ahpC*, *mshA*, and DRMs that confer resistance to other antibiotics, including *gyrA*, *gyrB*, *rrs*, and the *eis* promoter region (24).

As yet, there is no definitive tool for the prediction of drug resistance from sequencing data, and thus, we chose to use the classifications proposed by Miotto et al. in their systematic review (4). However, this does not include a number of DRMs identified elsewhere, including *inhA* -47 (11), *inhA* I194T (25), *katG* A110V (11, 26, 27), *katG* A541D (28), and those outside the RRDR of *rpoB* (25). Nevertheless, the standardized approach developed by Miotto et al. provided a consistent and solid foundation on which to classify our strains as either resistant or susceptible to RIF and INH. Our resistance-calling algorithm by necessity is relatively simple, since there were insufficient mutations in the 29 clinical samples used in this study to facilitate a more complex analysis. However, our workflow is sufficiently robust to incorporate a more sophisticated resistance-calling algorithm (e.g., by allocating weights to individual mutations to reflect their known contributions to drug resistance). This would not be computationally expensive and so could be run on the same laptop as the existing pipeline. Our command-line pipeline is customizable (e.g., to incorporate separate base calling or read mapping algorithms) and takes minutes to produce results on a midrange Linux or Mac laptop. While the number of samples used in this study was sufficiently small to allow individual inspection of alignments to identify DRMs, the resulting alignments did not require manual curation (with the exception of the systemic frame-shifts found on a single run). Thus, automating DRM calling within the pipeline should be a trivial task, further reducing the informatics expertise required to use our workflow.

### Challenges and future work.

Despite successfully showing the potential of our workflow to be used for the detection of mycobacterial DRMs, there are a number of outstanding issues to be addressed and potential improvements that could be made to maximize its utility, which are summarized in Table 5. Sample processing remains a significant challenge for all sputum-based TB diagnostic pathways. Methods that differentially lyse human cells prior to mycobacterial cells have shown promising results (13). Also, sputum collection has been made possible in community settings by low-cost tools like the “septum” sample pot, which allows a lysis buffer to be added via a valve after the lid is closed, for safe handling of potentially infectious samples in the community (29).

**TABLE 5:** Challenges for the development of our rapid TB diagnostic workflow

Challenge	Issue	Solution^*a*^
Sample processing	Insufficient mycobacterial DNA	Differential lysis of human/TB cells to maximize mycobacterial DNA extracted
Library prepn	Time/cost	Modify RPA primers with ONT proprietary chemistry to use ONT PCR sequencing kit
	Time/cost	Freeze-dry RPA primers and reagents to multiplex RPA assay
Sequencing	Cost/hardware	ONT SmidgION, which requires mobile phone rather than laptop/PC
	Cost	ONT Flongle flow cell, which has significantly lower costs than standard MinION flow cells
Scalability	Limited gene panel	Develop RPA primers for additional genes of interest

a ONT, Oxford Nanopore Technologies; RPA, recombinase polymerase amplification; Flongle, trade mark based on “flow cell dongle.”

A further limitation is the time required for nanopore sequencing library preparation and the cost of the reagents. However, using the PCR sequencing kit for library preparation rather than the ligation sequencing kit (both supplied by Oxford Nanopore Technologies) would reduce sample-to-answer time and also potentially reduce the cost of the workflow, although the RPA primers would require modification with Oxford Nanopore’s “rapid attachment chemistry.” Similarly, it would be possible to further simplify the RPA step by freeze-drying primers with reagents (30) and by multiplexing the RPAs (17, 31). This would further reduce the cost and time requirements of the workflow, as well as its deployability to resource-limited settings.

The rapidly evolving landscape of next-generation sequencing, and particularly nanopore sequencing, means that the costs of sequencing are falling year-on-year. For example, Flongle flow cells will allow much more affordable nanopore sequencing and could potentially drive down the price of our workflow further still. The SmidgION, a mobile phone-based sequencer that will soon be released by Oxford Nanopore Technologies, will also increase the deployability of sequencing.

One factor not addressed by this study is the potential for sequencing failure. The samples chosen for this study were all previously successfully sequenced by Illumina HiSeq, and we did not have access to clinical samples that had failed to generate genomes. However, in a separate study, we have found that samples that fail to sequence on the MinION invariably also fail to generate actionable sequence on the Illumina MiSeq, the determining factor being insufficient pathogen genomic material initially present in the clinical sample (data not shown). However, in contrast to competing sequencing platforms, the MinION has the potential for longer sequencing runs, analyzed in real time, to generate greater read depths that could mitigate this issue. We have shown that sufficient reads are generated within 90 s to accurately call DRMs from the clinical samples used in this study, but allowing the MinION to sequence for longer might enable the generation of reliable sequences for samples with lower concentrations of mycobacterial DNA. There is huge scope for automated analysis of nanopore sequencing data. Programs that can produce clinician-friendly reports will increase throughput and play an important role in bringing the technology to near-point-of-care settings. However, a relatively high level of expertise is still required to prepare samples for sequencing, and careful quality control of data also remains important.

Rather than an alternative to existing molecular diagnostics, we see our workflow providing a useful addition to TB health care provision to accurately identify DRMs in existing or high-risk TB patients as part of monitoring patient treatment and prognosis. In this way our workflow could be used to inform treatment, including the use of higher doses of antibiotics when lower-grade-resistance DRMs are identified.

## MATERIALS AND METHODS

### Nucleic acid amplification.

RPA primer design is described in the supplemental material. Custom RPA kits (TwistDx) tailored for the production of long amplicons were used. The TwistAmp basic kit was also used for comparison experiments. KOD Xtreme hot start DNA polymerase (Merck Millipore) was used in PCR experiments according to the manufacturer’s instructions. RPA, primer screen assays, PCR, purification of amplification products, and agarose gel electrophoresis are described in the supplemental material.

### MiSeq/Illumina sequencing.

MiSeq/Illumina library preparation and data analysis are described in the supplemental material. Briefly, libraries were generated using the Nextera XT DNA sample preparation kit (Illumina) using 1 ng DNA per sample. Following purification and sample library normalization, samples were pooled, loaded onto a MiSeq reagent kit (v2, 500 cycles), and sequenced on a MiSeq. Consensus sequences were generated from short reads using an in-house reference-based genome assembly pipeline. We applied a read depth cutoff of ≥20 reads to the final sequences. Sequence alignments to the H37Rv genome were performed using MAFFT version 7.305.

### Nanopore sequencing.

Library preparation and sequencing is described in detail in the supplemental material. Briefly, 16 µl of each RPA amplicon (*rpoB*, *katG*, and *inhA*) for each sample was combined, resulting in a total DNA mass of between 106.56 and 393.60 ng. DNA repair and end preparation was performed before a bead cleanup using AMPureXP beads and elution in water following two 70% ethanol wash steps. DNA was eluted with nuclease-free H_2_O. The native barcoding expansion 1-12 kit (SKU EXP-NBD104; Oxford Nanopore Technologies) was used to barcode each sample. A bead cleanup of the DNA was performed as described above. The DNA concentration was determined, and equimolar amounts of each barcoded sample were added to a clean Eppendorf tube, up to a total of 400 ng. The ligation sequencing kit (SKU SQK-LSK109; Oxford Nanopore Technologies) was used for adapter ligation, followed by a bead cleanup. Adapters were ligated using adapter mix (Oxford Nanopore Technologies). A bead cleanup was performed, and DNA was eluted in 13 µl elution buffer (both from Oxford Nanopore Technologies). The final sequencing library was prepared by mixing 50 ng eluted DNA with 37.5 µl sequencing buffer and 25.5 µl loading beads (both from Oxford Nanopore Technologies).

### Nanopore sequencing data analysis.

FASTQ files were generated for each run using MinKNOW (version 18.2.9; Oxford Nanopore Technologies Ltd.) and demultiplexed with Porechop (32) and qcat (version 1.01; Oxford Nanopore Technologies Ltd.). Reads were mapped to the M. tuberculosis H37Rv reference strain (GenBank accession number AL123456.3) with minimap2 (33), and consensus sequences and variants for each sample were generated using samtools (25) and custom in-house Perl scripts (available on request). Alignments were inspected using AliView (34), and statistical analysis was performed in R (35).

### Ethics statement.

Ethical approval for the Clinical Urine Sputum Blood Study (CUBS), as described by Cohen et al. (22), was granted on 13 April 2013 by the University of KwaZulu-Natal Biomedical Research Ethics Committee with reference number BE022/13 and approved one-off specimen collection to allow the phenotypic and genotypic characterization of M. tuberculosis isolates.

### Participant recruitment.

Inclusion criteria for CUBS included an age of 18 years or more and written informed consent (22). Other than failure to meet inclusion criteria, exclusion criteria included any psychological or psychiatric condition preventing the giving of informed consent. All participants included in this study were recruited from King George V Hospital between 24 May and 23 August 2013. Participants gave sputum samples at the same time as enrollment in TB treatment. M. tuberculosis was isolated using MGIT culture and culture on 7H11 medium. DST was by performed by critical concentration for first-line (including RIF [1 μg/ml] and INH [0.2 and 1 μg/ml]) and second-line antitubercular drugs on Middlebrook 7H11.

We performed a sample size estimation to verify the number of samples sufficient for prediction of drug resistance (see the supplemental material). Among the participants who gave the 29 samples, the median age was 36 (IQR, 31 to 42), the cohort was 75.86% male, and 89.7% were living with HIV. Most (89.7%) were smear positive. Phenotypic DST results showed that 25 of the isolated strains (86.2%) were INH resistant (INH R) and 27 (93.1%) were RIF resistant (RIF R). Most (65.5%) strains were classed as MDR, but 27.7% were classed as XDR, 6.9% were susceptible to all antibiotics tested, and another 6.9% were classed as monoresistant (resistant only to RIF). The genotypic results for drug susceptibility were highly concordant with the phenotypic results, with no differences found in calling resistance to either INH or RIF (see the supplemental material).

### Calculation of sensitivity and specificity of DR prediction.

Statistical analyses, including calculation of sensitivity and specificity, were performed using the R statistical computing language. The exact binomial method was used to calculate 95% confidence intervals.

### Data availability.

The unprocessed sequencing data generated in this study are openly available in the European Nucleotide Archive (ENA, https://www.ebi.ac.uk/ena) under study accession number PRJEB43981, with sample accession numbers ERS6142037 to ERS6142065, ERS6146501 to ERS6146506, and ERS6146515 to ERS6146526. Processed data are freely available on request to the corresponding author.
