# Supplementary material for: A Rapid Drug Resistance Genotyping Workflow for Mycobacterium tuberculosis, Using Targeted Isothermal Amplification and Nanopore Sequencing
Source: Microbiol Spectr. 2021 Nov 24;9(3):e00610-21. doi: 10.1128/Spectrum.00610-21 (PMC8612157; doi:10.1128/Spectrum.00610-21)
Supplement: SUPPLEMENTAL FILE 1 — Supplemental material. Download SPECTRUM00610-21_Supp_1_seq1.pdf, PDF file, 1.6 MB [file spectrum00610-21_supp_1_seq1.pdf]

Supplemental Material

Supplemental methods

Primer design
RPA primers were designed in Genome Compiler[1] against the H37Rv reference genome.[2] For the *rpoB* RPA assay, the RRDR was selected to be the midpoint of the amplicon, and 700 bp either side of codon 450 (position 761155) was used in order to design the assay. For the *katG* RPA assay, we aimed to cover from codon 541 (position 2,154,488) to codon 110 (position 2,155,783). For the *inhA* RPA assay, we aimed to cover from -47 (position 1,673,393) to codon 194 (position 1,674,782). The *rpoB* PCR primers were designed to anneal slightly outside of those of the final *rpoB* RPA assay, so the entire RPA amplicon was included in the PCR amplicon. This region was then entered into Primer-BLAST[3] to design PCR primers.

Nucleic acid amplification by RPA
Up to 10 µL template DNA was added, adjusted using nuclease-free H_2O. Reactions were incubated at 37°C for 90 minutes unless otherwise stated in a shaking incubator set to 154 rpm. Tubes were heated to 85°C for 5 minutes to terminate the reaction. Unless otherwise stated, 10 µL of 5 ng/µL H37Rv DNA (50 ng total) was added to each RPA assay. For the limit of detection study, serial dilutions of H37Rv DNA were made before adding to reactions. For generation of RPA amplicons for sequencing, 10 µL of 5 ng/µL DNA sample (50 ng total) was added to each RPA assay.

Primer screen for RPA assays
H37Rv Bacterial qDNA PCR control (Advanced Biotechnologies Inc) was used as template DNA at a final concentration of 65 copies/µL. Each primer combination for each RPA assay was used in three independent repeats. Resulting amplicons were analysed by agarose gel electrophoresis, followed by Illumina sequencing.

Nucleic acid amplification by PCR
KOD Xtreme™ Hot Start DNA Polymerase (Merck Millipore) was used in PCR experiments according to the manufacturer's instructions. Reactions were made up to 50 µL, the final concentration for each dNTP was 400 µM, the final reaction buffer was 1X, the final primer concentration was 0.3 µM and the final polymerase concentration was 0.02 U/µL. As for RPA, 10 µL of 5 ng/µL DNA sample (50 ng total) was added to each PCR reaction. The thermal cycling was performed on a Bio-Rad T100 Thermo Cycler and consisted of an initial denaturation of 94°C for two minutes, followed by 35 cycles of 98°C for 20 seconds, 53°C for 30 seconds and 68°C for 40 seconds. A final extension of 68°C for ten minutes was then followed by a hold at 4°C.

Purification of RPA and PCR amplification products
Amplification products were purified using either QIAquick PCR Purification Kit (QIAGEN) or GeneJET PCR Purification Kit (Thermo Fisher Scientific) according to the manufacturer's instructions and eluted in 50 µL nuclease-free H_2O.

Agarose gel electrophoresis
1% agarose gels were made using either 1X TBE or 1X TAE and stained with SYBR Safe. Molecular markers were either 1 Kb Plus DNA Ladder or O'GeneRuler™ DNA Ladder Mix, and samples were loaded with DNA Loading Dye or BlueJuice Gel Loading Buffer (both Thermo Fisher Scientific). Gels were run at run at 50V for 10 minutes, then 100V for up to 60 minutes and imaged using either an E-Gel Imager with a Blue Light Base (Thermo Fisher Scientific) or a ChemiDoc XRS System (Bio-Rad). Gel band intensities were calculated using ImageJ[4].

MiSeq/Illumina library preparation
The total DNA concentration of samples was determined using the Qubit HS DNA assay (Invitrogen). Library preparations were generated using the Nextera XT DNA sample preparation kit (Illumina) according to the manufacturer's instructions using a total of 1 ng DNA input per sample. Tagged samples were purified using 30µl AMPure XP beads (Beckman Coulter). Following sample library normalisation, samples were pooled and loaded onto a MiSeq reagent kit (V2 500 cycles) and sequenced on a MiSeq. A Phi-X control was added at a final concentration of 0.1pM.

MiSeq/Illumina sequencing data analysis
Consensus sequences were generated from short reads using an in-house reference-based genome assembly pipeline consisting of custom Perl scripts alongside Trimmomatic (v0.33), SMALT (v0.7.6), SAMtools (v1.2), IVA (v1.0.9) and lastz (v1.03.54). We applied a read depth cut-off of ≥20 reads to the final sequences. Sequence alignments to the H37Rv genome were performed using MAFFT v7.305.

Nanopore sequencing library preparation
Following purification, 16 µL of each RPA amplicon (*rpoB, katG* and *inhA)* for each sample were combined, resulting in a total DNA mass of between 106.56 and 393.60 ng, as measured using the Qubit HS DNA assay (Thermo Fisher Scientific). DNA repair and end prep for each sample was performed using 3.5 µL NEBNext FFPE DNA Repair Buffer, 2 µL NEBNext FFPE DNA Repair Mix, 3.5 µL Ultra II End-prep reaction buffer and 3 µL Ultra II End-prep enzyme mix (New England Biolabs) following the manufacturer's protocol, in thin-walled PCR tubes before incubating at 20°C for 15 minutes, followed by 65°C for 15 minutes.

A bead cleanup of the DNA was then performed with 108 µL AMPureXP beads and elution in water following two 70% ethanol wash steps. After five minutes of incubation at room temperature (RT), beads were washed twice with 200 µL 70% ethanol. DNA was eluted with 25 µL nuclease-free H_2O.

The Native Barcoding Expansion 1-12 Kit (EXP-NBD104, Oxford Nanopore Technologies) was used to barcode each sample. A total of 12 samples were run on each flow cell, and a single barcode per sample was used; barcodes were ligated with µL Blunt/TA Ligase Master Mix (New England Biolabs). To ligate barcodes, 22.5 µL end-prepped DNA and 25 µL Blunt/TA Ligase Master Mix (New England Biolabs) were added to 2.5 µL of the native barcode and incubated at RT for one hour.

A bead cleanup of the DNA was performed as previously, using 90 µL AMPureXP beads and 26 µL nuclease-free H_2O for elution. DNA concentration was determined using the Qubit HS DNA assay, and equimolar amounts of each barcoded sample were added to a clean 1.5 mL Eppendorf, up to a total of 400 ng.

The Ligation Sequencing Kit (SKU: SQK-LSK109, Oxford Nanopore Technologies) was used for adapter ligation according to the maufacturer's instructions, followed by a bead cleanup as before using 180 µL AMPureXP beads. Beads were then washed twice with 250 µL S Fragment Buffer and DNA eluted in 13 µL Elution Buffer (both Oxford Nanopore Technologies). To ligate adapters, 65 µL pooled barcoded sample was added to 5 µL Adapter Mix (Oxford Nanopore Technologies), 20 µL NEBNext Quick Ligation Reaction Buffer (5X) and 10 µL Quick T4 DNA ligase (both New England Biolabs, Massachusetts, United States) were incubated at RT for 10 minutes.

A bead cleanup was performed as before using 180 µL AMPureXP beads. Beads were washed twice with 250 µL S Fragment Buffer. DNA was eluted in 13 µL Elution Buffer (both Oxford Nanopore Technologies). The final sequencing library was prepared by mixing 50 ng eluted DNA with 37.5 µL Sequencing Buffer and 25.5 µL Loading Beads (both Oxford Nanopore Technologies).

Nanopore sequencing

A SPOT ON Flow Cell MK 1 R9.4 Version (SKU: FLO-MIN106D, Oxford Nanopore Technologies) was used for sequencing and run using the MinKNOW Software Version 18.12.9. The flow cell was placed in the MinION and following quality control checks the flow cell was primed as recommended by the manufacturer and the DNA library was loaded. The sequencing run was set for three hours, with a MUX scan every 60 minutes and basecalling was set to offline.

Sample size estimation

We had access to genomic DNA extracts for 29 of the original 30 strains collected during the Collection of Urine, Blood and Sputum (CUBS) Study, as described by Cohen et al.[5] We calculated if this number would be sufficient for us to assess the diagnostic accuracy of our workflow. The number of RIF- and INH-resistant and susceptible strains was known from previous phenotypic DST, and therefore the 'prevalence' of resistance could be calculated. Twenty-seven of the 29 samples were RIF resistant, giving a prevalence of 93.1%. 25 of the 29 samples were INH resistant, giving a prevalence of 86.2%. We used sensitivity values for calling rifampicin and isoniazid resistance given in the Miotto et al supplementary table S10.3 for mutations of High and Moderate confidence levels only.[6] These were 90.4% and 78.3% for RIF and INH respectively. We used Buderer's formula for sample size calculation, based on sensitivity:

$$\text{Sample size (n) based on sensitivity} = \frac{Z^2_{1-\alpha/2} \times S_N \times (1 - S_N)}{L_2 \times \text{Prevalence}}$$

Where n is the required sample size, SN is the anticipated sensitivity, α is the size of the critical region, with 1-α as the confidence level, Z1-α/2 is the standard normal deviate corresponding to the specified size of the critical region (α), and L is the absolute precision desired on either side (half-width of the confidence interval). For 95% confidence intervals, L value was 1.96. Our desired precision was 15%. The resulting sample size estimates were 15.9 for RIF and 32.9 for INH. Since the actual number of samples we had access to was 29, we deemed this sufficient to proceed with the study.

Participants in the CUBS study

Of the 29 clinical samples used, the median age was 36 (IQ 31-42), the cohort was 75.9% male and 89.7% were living with HIV. Most (89.7%) were sputum smear microscopy-positive.

Sample processing, microbiological culture and DNA extraction

Sputum samples were given at the same time as enrolment TB treatment. *M. tuberculosis* was isolated using MGIT culture and culture on 7H11 media. Genomic DNA extraction was previously performed by Cohen et al[5] using the NaCl-CTAB method[7] from either Mycobacteria Growth Indicator Tube (MGIT) samples (34.5%) or directly from single colony isolations (65.5%).

Mycobacterial strains in the CUBS study

Drug susceptibility testing (DST) was by performed by critical concentration for first (including RIF (1 µg/mL) and INH (0.2 and 1 µg/mL) and second-line antitubercular drugs on Middlebrook 7H11. Phenotypic DST results showed that 25 of the isolated strains (86.2%) were INH R, and 27 (93.1%) were RIF R. Most (65.5%) strains were classed as MDR, but 27.7% were classed as XDR, 6.9% were susceptible to all antibiotics tested and another 6.9% were classed as mono resistant (only to RIF).[5] The genotypic results for drug susceptibility were highly concordant with the phenotypic results, with no differences found in calling resistance to either INH or RIF.

Table S1. RPA and PCR primers used in this study. All RPA primers used in the initial primer screen are listed, with primers selected for further use highlighted in grey. Primer sequences were compared to the sequences of clinical strains of *M. tuberculosis* across four databases to investigate the presence of known variants.

					Known variants within primer sequence detected in clinical strains using the databases listed below			
**Primer**	**Sequence (5' - 3')**	**Length (bp)**	**Melting temp. (°C)**	**GC content (%)**	**TBDream[8]**	**TBVar (India)[9]**	**polyTB (LSHTM Malawi)[10]**	**GMTV (Russia)[11]**
rpoB RPA F1	ATCATGCGATCGACGCTG GAGAAGGACAACACC	33	67	55	None	760566 1x C	None	760566 x1 C
rpoB RPA F2	GCAGACGCTGTTGGAAAA CTTGTTCTTCAAGGA	33	63	45	None	None	None	None
rpoB RPA F3	TGGCCCGCGTCGGTCGCT ATAAGGTCAACAAGA	33	68	58	None	None	None	None
rpoB RPA R1	GACCAGGATGTCCCCGTC GCGAACCTCGGCACC	33	73	70	None	None	None	None
rpoB RPA R2	GCACCTCGTCGGAGATGT TCGGGATGTCGCGGG	33	72	67	None	None	None	None
rpoB RPA R3	TGATCTCCTCCGCACCCAG CTTGGTGTCGCGAG	33	71	64	None	None	None	None
rpoB RPA R4	AGGTGAGCACGTCCTCTT CGACCAGGCGGTTGGA	34	70	62	None	None	None	None
katG RPA F1	GGCTCCAGCACGGCAAAG GATTCCACGTCG	30	68	63	587: CTG/ATG; 587: CTG/CCG; 589:CCC/ACC	None	None	581: GAC/GAT 3x; 586: GTG/ATG 2x
katG RPA F2	GGCGGCACAGCCACCGAG CACGACGAGGT	29	71	72	551: GCC/GGC	None	None	None
katG RPA F3	CGTGATGTTGTGGCCAGC CGCCTTTGCTGCT	31	68	61	556: delC	None	None	None
katG RPA R1	ACCTCGCAGCCGTGGTGG CCCGCCGACTAC	30	73	73	88: CAG/CGG; 90: TGG/TGA; 91: TGG/CGG; 94:GAC/GGC; 94: GAC/GCC	2155832 C/T SNP 0.2%	None	86: ACC/CCC 3x; 90 TGG/AGG 1x;
katG RPA R2	GAGGAAGTGATGACCACC TCGCAGCCGTG	29	67	62	84: ATG/ATA; 85: ACC/CCC	None	None	86: ACC/CCC 3x; 90 TGG/AGG 1x;
katG RPA R3	GGCCACTACGGGCCGCTG TTTATCCGGATGG	31	70	65	96: GGC/TGC; 99: GGG/GAG; 104: CGG/CTG; 104: CGG/CAG; 106: GCG/GTG	None	None	99 GGG/GAG 2x; 104: CGG/TGG 1x
inhA RPA F1	GTGCTGAGTCACACCGAC AAACGTCACGAG	30	66	57	n/a	1673338: G/A 0.43%	None	1673338: G/A 2x
inhA RPA F2	ACGAGCGTAACCCCAGTG CGAAAGTTCCCG	30	67	60	n/a	None	None	1673346: C/G 1x; 1673349: G/C 4x;
inhA RPA F3	GCGTAACCCCAGTGCGAA AGTTCCCGCCGG	30	70	67	n/a	1673338: G/A 0.43%	None	1673338: G/A 0.43%; 1673346: C/G 1x; 1673349: G/C 4x;
inhA RPA F4	ACGTCACGAGCGTAACCC CAGTGCGAAAGTT	31	66	55	n/a	1673338: G/A 0.43%	None	1673338: G/A 0.43%; 1673346: C/G 1x; 1673349: G/C 4x;
inhA RPA R1	GAGCGCACCGCCGACGAT CGCACTCATC	28	69	68	None	None	None	None
inhA RPA R2	CTGGCTGCCGGCGACCAC GGGTGACATCATCTA	33	71	64	None	None	None	*1674952: CCG/GCG 91x; 1674966: GGT/GGG 33x*
inhA RPA R3	GAGCGCGCTGATCCCAGC CCTCCTCGAGCA	30	71	70	aa217: Glu/Asp	None	None	1674859: GAG/TAG 1x
rpoB PCR F1	ACGCTGGAGAAGGACAAC AC	20	54	55	None	None	None	None
rpoB PCR R1	TCGATGTGGATCGAGGTG AG	20	54	55	None	None	None	762039 1xT

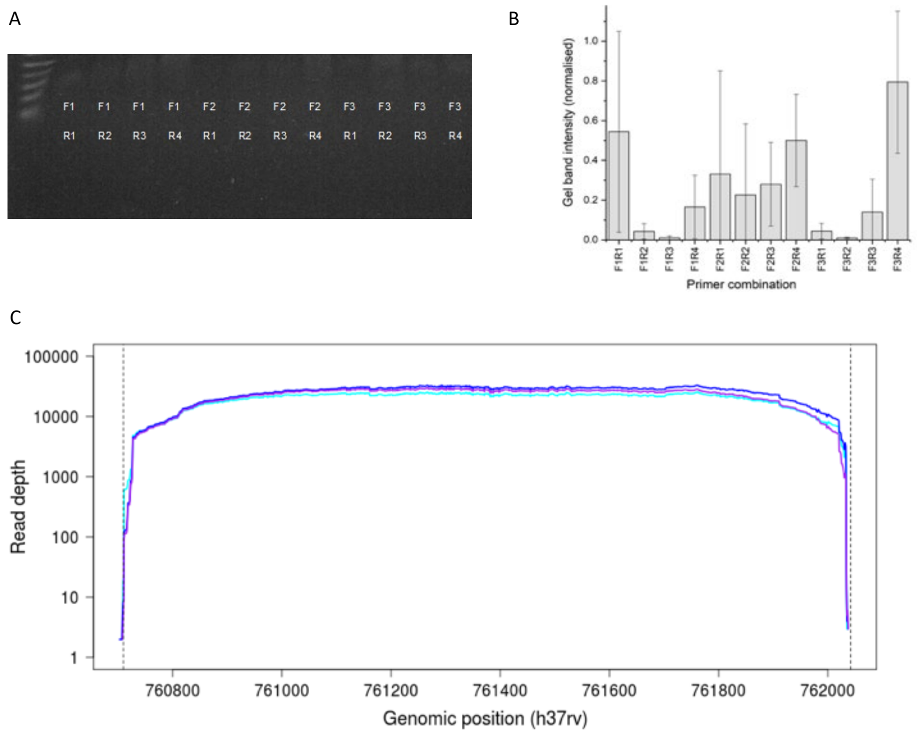

Figure S1. Primer screen results for *rpoB* RPA assay. (A) Representative 1% agarose gel, stained with SYBR Safe, of purified RPA amplicons for each primer combination. (B) Average gel band intensities for each RPA amplicon were calculated using ImageJ software (n=3). The primer combination that gave the amplicon with the highest intensity, and therefore the highest yield, was F3 and R4. (C) Illumina sequencing results for the *rpoB* RPA amplicon generated using primers F3 and R4.

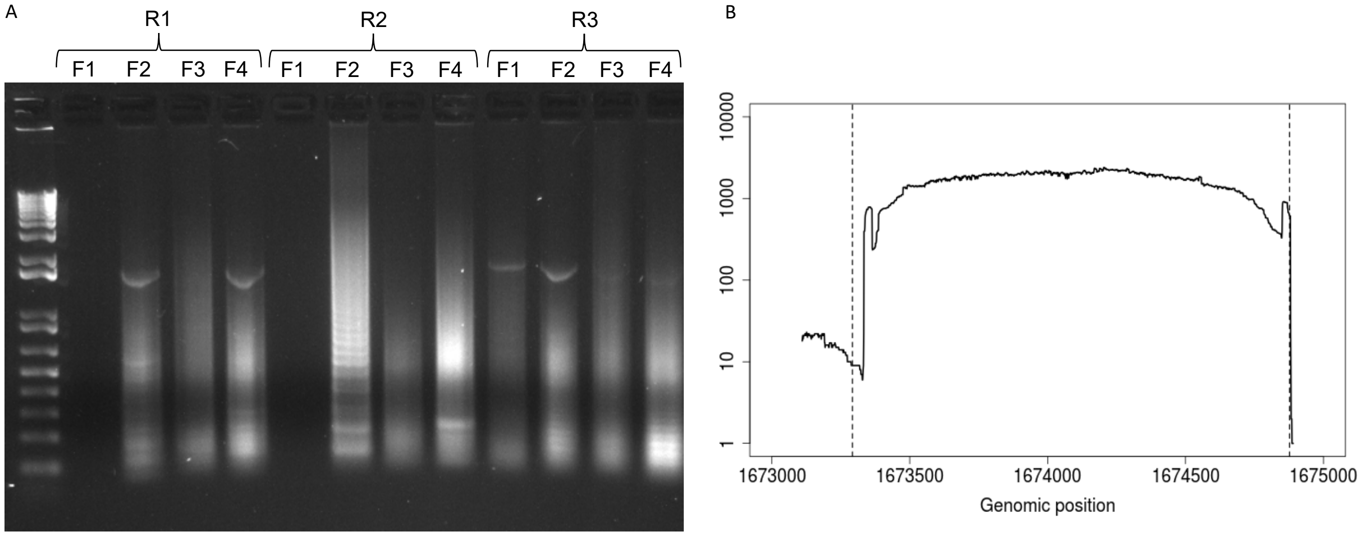

Figure S2. Primer screen for the *inhA* RPA assay. (A) 1% agarose gel of purified RPA products for each primer combination. (B) Illumina sequencing coverage plot for the product generated by the *inhA* RPA primers F2 and R3.

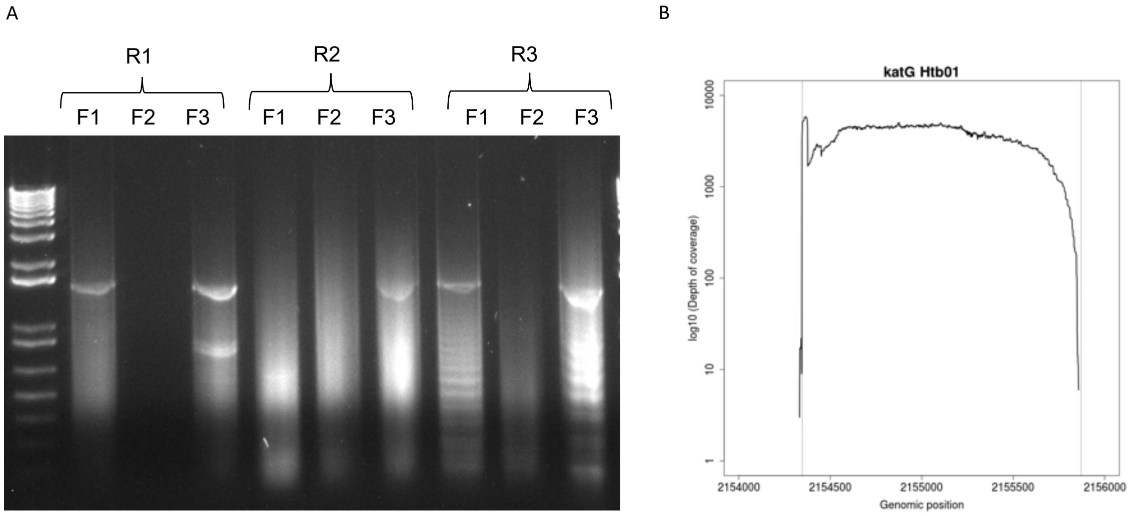

Figure S3. Primer screen for the *katG* RPA assay. (A) 1% agarose gel of purified RPA products for each primer combination. (B) Illumina sequencing coverage plot for the amplicon produced by *katG* RPA primers F1 and R1.

Table S2. Final primers and resulting amplicons used for sequencing, and their locations within the H37Rv genome.

**Amplicon**	**Size (bp)**	**Forward primer**	**Reverse primer**	**Start locus**	**End locus**
rpoB RPA	1,323	*rpoB* RPA F3	*rpoB* RPA R4	760,711	762,033
katG RPA	1,511	*katG* RPA F1	*katG* RPA R1	2,154,346	2,155,856
inhA RPA	1,547	*inhA* RPA F2	*inhA* RPA R3	1,673,334	1,674,880
rpoB PCR	1,478	*rpoB* PCR F1	*rpoB* PCR R1	760,569	762,046

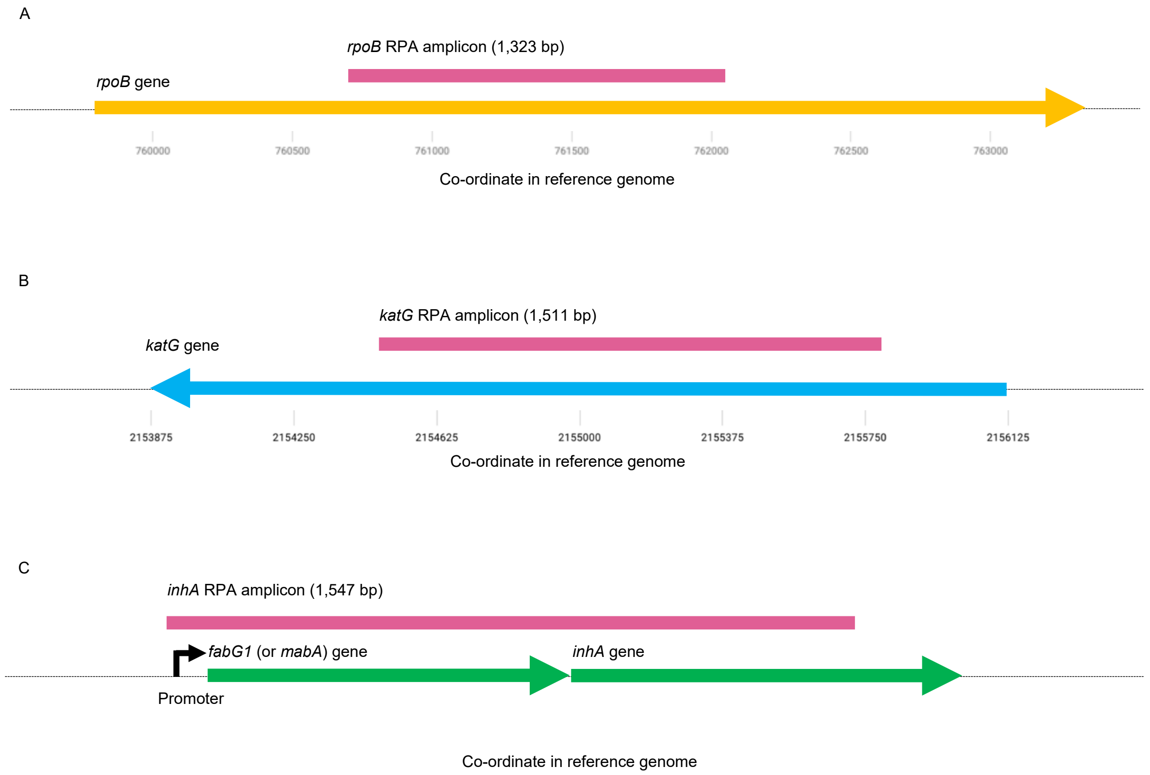

Figure S4. Schematic of RPA amplicon locations for the (A) *rpoB*, (B) *katG* and (C) *inhA* gene regions in reference to the H37Rv genome.

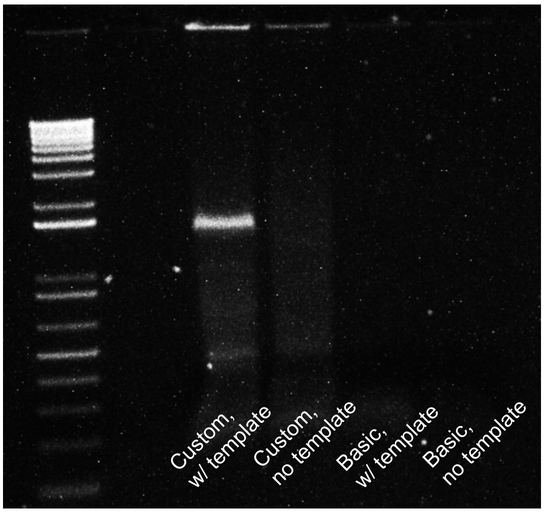

Figure S5. 1% agarose gel showing products of the *rpoB* RPA assay using the custom kit (optimised for the generation of amplicons of 1.5 kb) and basic kit (both TwistDx).

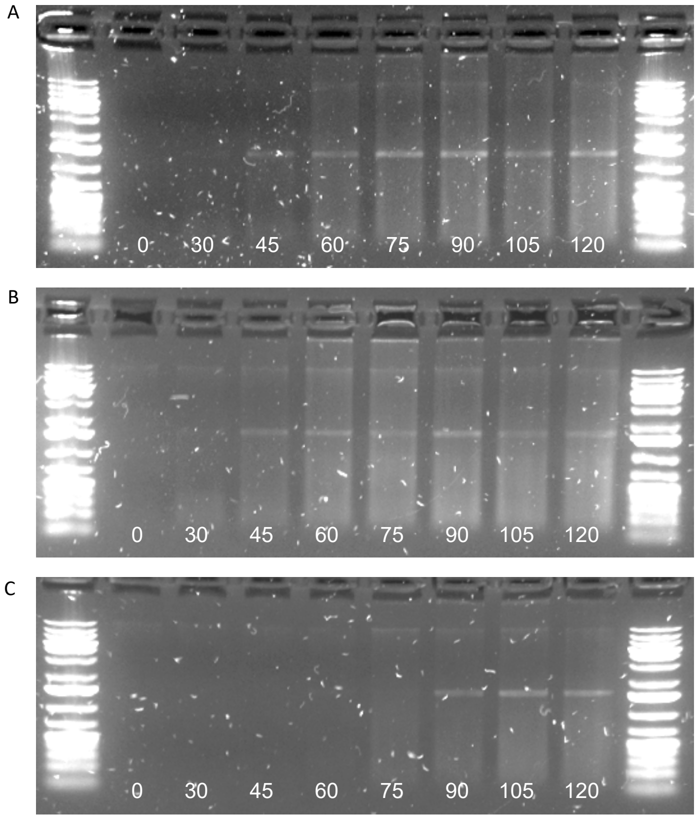

Figure S6. Assessment of RPA time requirements. 1% agarose gel for the (A) *rpoB*, (B) *inhA* and (C) *katG* RPA assays, with each lane showing products after inactivation following either 0, 30, 45, 60, 75, 90, 105 or 120 minutes of incubation time.

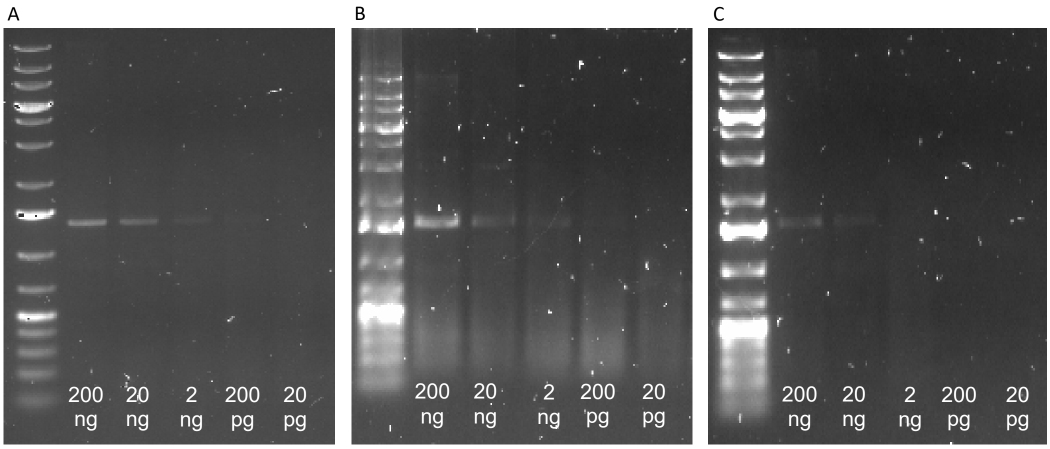

Figure S7. Assessment of required genomic DNA concentration for each RPA assay. 1% agarose gel showing products of the (A) *rpoB*, (B) *inhA* and (C) *katG* RPA assays using a range of concentrations of H37Rv DNA, including 200 ng, 20 ng, 2 ng, 200 pg, and 20 pg in 50 µL total RPA volume.

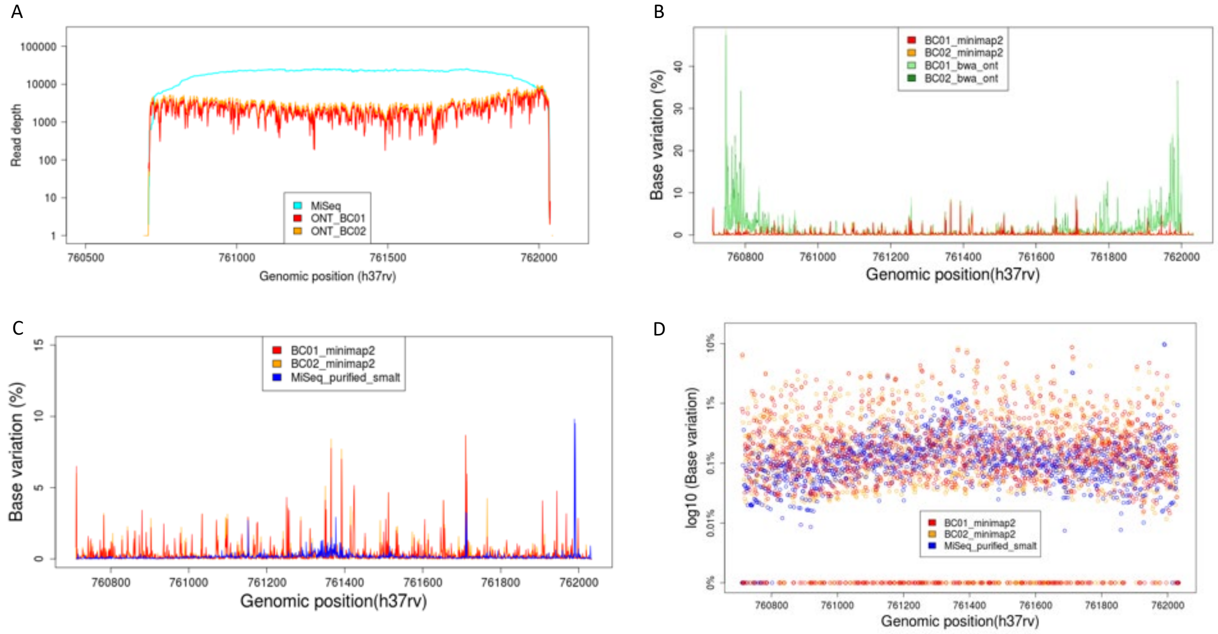

Figure S8. Comparison of nanopore and Illumina sequencing results for the *rpoB* RPA amplicon from reference gDNA after mapping to the H37Rv genome. The nanopore run was 30 minutes long, whereas the Illumina MiSeq run was 48 hours long. Read depth of coverage (A) and base variation (B-D) are shown across the region of interest within the *rpoB* gene. (A) Depth of coverage within the *rpoB* gene is shown for one MiSeq and two nanopore samples after mapping with smalt and Minimap2 respectively. (B) The proportion of nanopore reads mapping with differing nucleotides to H37Rv reference is shown after mapping with Minimap2 and bwa-ont: unexpectedly high variation at the ends of each amplicon corresponds to less accurate read mapping by bwa-ont. Observed sequence variation between MiSeq and nanopore platforms was compared on a linear (C) and log-scale (D). Variation on the MiSeq platform corresponds closely to Illumina's 0.1% error rate across the amplicon, whereas nanopore variation is more broadly spread. Approximately 15% of nanopore positions show no sequence variation at all (remarkable, given the read depth of ~1000 reads), whilst others show variation between 1-10%. As similar variation is not seen within the MiSeq reads we presume these are systematic base-calling errors. In time, improvements in base-calling algorithms will reduce the number of these errors, but calling low-level variants using nanopore sequencing may be unreliable at present.

Table S3. TB strains used for sequencing, as studied by Cohen et al.[5]

Study no.	Strain	Age	Sex	HIV Status	DNA isolation	INH DST result	RIF DST result
1	TKK_05MA_0004	18	F	N	MGIT	R	R
2	TKK_05MA_0009	37	M	P	MGIT	R	R
3	TKK_05MA_0033	28	F	P	MGIT	R	R
4	TKK_05MA_0035	37	M	P	MGIT	R	R
5	TKK_05MA_0037	24	F	P	MGIT	R	R
6	TKK_05MA_0040	34	M	P	MGIT	R	R
7	TKK_05MA_0051	21	F	N	MGIT	R	R
8	TKK_05MA_2005	29	M	P	MGIT	R	R
9	TKK_05MA_2008	43	M	P	MGIT	R	R
10	TKK_05MA_2015	43	M	P	MGIT	R	R
11	TKK_05SA_0010	47	M	P	SCI	R	R
12	TKK_05SA_0011	27	M	N	SCI	R	R
13	TKK_05SA_0014	39	M	P	SCI	R	R
14	TKK_05SA_0016	40	F	P	SCI	R	R
15	TKK_05SA_0018	49	M	P	SCI	R	R
16	TKK_05SA_0019	31	M	P	SCI	S	R
17	TKK_05SA_0020	31	M	P	SCI	R	R
18	TKK_05SA_0021	30	M	P	SCI	S	S
19	TKK_05SA_0024	36	M	P	SCI	S	S
20	TKK_05SA_0025	42	M	P	SCI	R	R
21	TKK_05SA_0042	38	M	P	SCI	R	R
22	TKK_05SA_0043	38	M	P	SCI	R	R
23	TKK_05SA_0044	32	M	P	SCI	R	R
24	TKK_05SA_0046	31	F	P	SCI	S	R
25	TKK_05SA_0048	52	M	P	SCI	R	R
26	TKK_05SA_0050	44	F	P	SCI	R	R
27	TKK_05SA_0052	44	M	P	SCI	R	R
28	TKK_05SA_0054	36	M	P	SCI	R	R
29	TKK_05SA_0055	35	M	P	SCI	R	R

INH: isoniazid; RIF: rifampicin; DST: drug-susceptibility testing; N: Negative; P: Positive; MGIT: Mycobacteria Growth Indicator Tube; SCI: single colony isolation; R: resistant; S: susceptible.

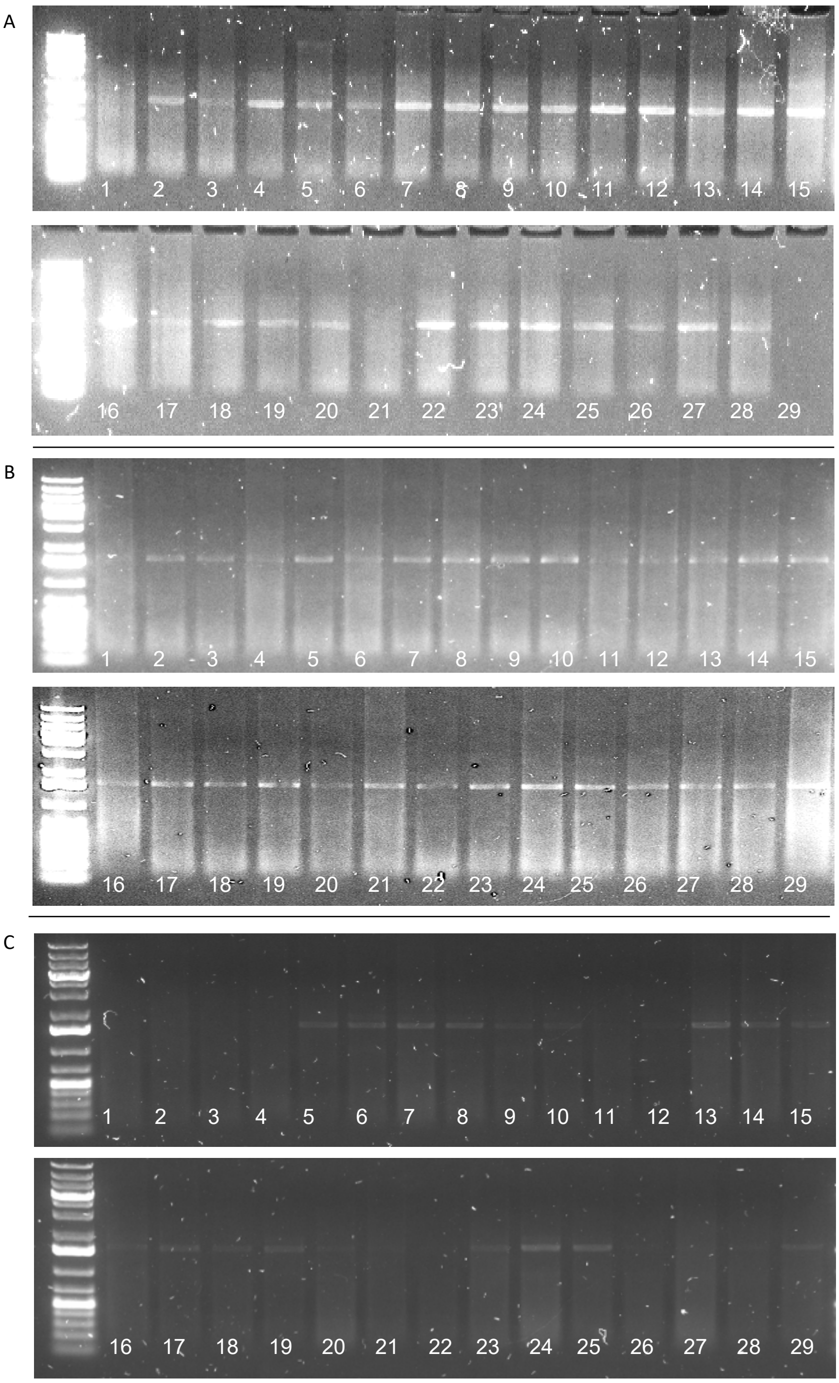

Figure S9. 1% agarose gel showing the products of the (A) *rpoB*, (B) *inhA* and (C) *katG* RPA assays for all 29 CUBS samples, prior to pooling and nanopore sequencing library preparation.

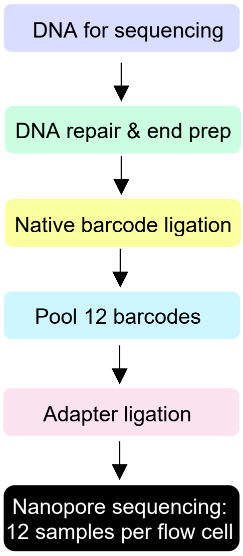

Figure S10. Nanopore sequencing library preparation workflow using the ligation sequencing kit and native barcoding kit.

Table S4. Flow cell QC for each of the four flow cells used for nanopore sequencing.

	**Number of pores available for each mux scan**		
**Flow cell**	1	2	3
1	1429	1534	967
2	1344	1381	1348
3	851	1296	1101
4	1107	1373	1407
Mean	1100.667	1350	1285.33

Table S5. The number of reads for each of the four flow cells used for nanopore sequencing.

Flow cell	Total reads	Total reads mapping to H37Rv	% barcoded reads	Number of non-barcoded reads	Mean reads per barcode	Median reads per barcode
1*	1273686.9	60150.9	65.2	208748.0	357853.7	613463.5
2	2344612.0	97082.0	86.4	319029.0	195384.3	193367.5
3	1945615.0	128166.0	90.9	176648.0	137338.0	154946.5
4	1059098.0	151649.0	90.5	100902.0	88258.2	105610.5
Mean	1655753.0	109262.0	83.2	201331.8	194708.6	266847.0

*Five samples for another study were run on Flow cell 1, and the remaining seven samples were from this study. We have adjusted the values given here proportionally for Flow cell 1.

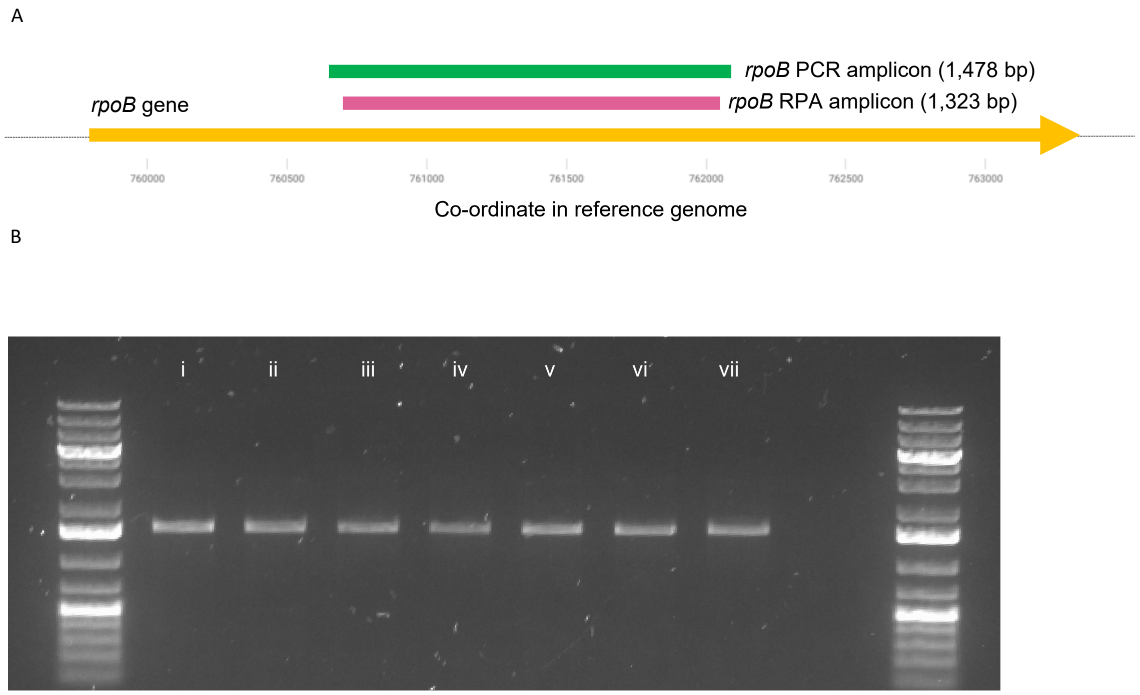

Figure S11. (A) Overview of the PCR amplicon for *rpoB* in relation to the RPA amplicon. (B) 1% agarose gel showing the products of the *rpoB* high fidelity PCR assay following purification. These were used to evaluate the fidelity of RPA. Due to space constraints on the flow cell, only five samples were sequenced. (i) TKK_05MA_0051, (ii) TKK_05SA_0011, (iii) TKK_05SA_0016, (iv) TKK_05SA_0018, (v) TKK_05SA_0048, (vi) TKK_05SA_0046 and (vii) TKK_05SA_0010.

Table S6. Number of reads from nanopore sequencing for the fidelity study samples.

	Number of reads	
**Sample ID**	**PCR amplicon**	**RPA amplicon**
TKK_05MA_0051	33336	117655
TKK_05SA_0011	26223	118837
TKK_05SA_0016	24794	100881
TKK_05SA_0018	27779	82732
TKK_05SA_0048	22964	120059
Mean no. reads	27019.2	108032.8

Table S7. Amplicon lengths and overall median read depth for fidelity study samples.

	**Mean amplicon length**		**Median amplicon length**		**Overall median read depth**	**% sequence with depth <20 reads**
	**No cut-off**	**Depth>=20**	**No cut-off**	**Depth>=20**		
***rpoB* PCR**	1506.80	1479.80	1503.00	1480.00	10155.00	1.79
***rpoB* RPA**	1491.00	1343.00	1478.00	1327.00	4365.00	9.93

Table S8. Nanopore sequencing results for the pooled RPA amplicons.

	**Mean amplicon length**		**Median amplicon length**		**Overall read depth**	**% sequence with depth <20 reads**
	**No cut-off**	**Depth>=20**	**No cut-off**	**Depth>=20**		
rpoB	1416.00	1321.82	1432.00	1325.00	1032.25	7.47
katG	1543.25	1494.07	1523.50	1510.00	253.50	0.89
inhA	1574.18	1547.21	1555.00	1547.00	557.75	0.51

Table S9. Details of total reads and reads mapping to H37Rv for each CUBS strain, including the number and percentage of reads for each RPA amplicon, and their median read depths.

				Number of reads mapping to:					% of H37Rv reads mapping to:			Median read depths		
**CUBS_ID**	**Total reads**	**Number of reads mapped to H37Rv**	**% reads mapped to H37Rv**	***rpoB***	***katG***	***inhA***	**All amplicons**	**% H37Rv reads mapping to amplicons**	***rpoB***	***katG***	***inhA***	***rpoB***	***katG***	***inhA***
TKK_05SA_0014	90815	5156	5.7	1348	1622	1790	4760	92.3	28.3	34.1	37.6	444.5	378	312
TKK_05SA_0016	86644	7602	8.8	2921	993	2314	6228	81.9	46.9	15.9	37.2	865	220	554
TKK_05SA_0018	86514	6948	8.0	4027	924	1614	6565	94.5	61.3	14.1	24.6	1372	215	454
TKK_05SA_0019	76023	2875	3.8	1478	500	519	2497	86.9	59.2	20.0	20.8	491.5	105	106
TKK_05SA_0020	77748	5217	6.7	2633	564	1419	4616	88.5	57.0	12.2	30.7	829.5	174	278
TKK_05SA_0021	102589	2971	2.9	483	536	1627	2646	89.1	18.3	20.3	61.5	159	146	386
TKK_05SA_0024	78776	4305	5.5	1295	820	1764	3879	90.1	33.4	21.1	45.5	426	190	395
TKK_05MA_0004	213171	3899	1.8	115	599	2028	2742	70.3	4.2	21.8	74.0	35	69	257
TKK_05MA_0033	264068	4970	1.9	324	214	3502	4040	81.3	8.0	5.3	86.7	67	62	540
TKK_05MA_0037	175968	13059	7.4	2526	2384	6209	11119	85.1	22.7	21.4	55.8	955	596	811
TKK_05MA_0040	194518	5855	3.0	1606	1992	1858	5456	93.2	29.4	36.5	34.1	591	620	317
TKK_05MA_0051	181051	8263	4.6	3407	1486	2763	7656	92.7	44.5	19.4	36.1	1468.5	565	604
TKK_05MA_2005	202394	9310	4.6	3072	2358	3258	8688	93.3	35.4	27.1	37.5	1261	784	839
TKK_05MA_2008	194009	10882	5.6	4071	1503	4331	9905	91.0	41.1	15.2	43.7	1694.5	370	1055
TKK_05MA_2015	192726	15586	8.1	3092	1711	9105	13908	89.2	22.2	12.3	65.5	1328	344	1633
TKK_05SA_0011	172647	9250	5.4	5255	736	2193	8184	88.5	64.2	9.0	26.8	2255	185	459
TKK_05SA_0025	196942	6039	3.1	1797	853	2372	5022	83.2	35.8	17.0	47.2	735.5	239	332
TKK_05SA_0042	171989	5153	3.0	1166	449	2801	4416	85.7	26.4	10.2	63.4	500	122	641
TKK_05SA_0043	185129	4792	2.6	195	336	3371	3902	81.4	5.0	8.6	86.4	69	103	603
TKK_05MA_0009	174337	8558	4.9	3372	601	3074	7047	82.3	47.9	8.5	43.6	1314.5	30	467
TKK_05MA_0035	177994	5271	3.0	3849	87	775	4711	89.4	81.7	1.8	16.5	1653.5	26	245
TKK_05SA_0044	131581	6754	5.1	2982	963	2366	6311	93.4	47.3	15.3	37.5	1385	330	629
TKK_05SA_0046	159493	8188	5.1	2033	2183	2940	7156	87.4	28.4	30.5	41.1	906.5	815	887
TKK_05SA_0048	140411	9068	6.5	2791	1786	3793	8370	92.3	33.3	21.3	45.3	1198	690	881
TKK_05SA_0050	204286	7750	3.8	2494	354	4447	7295	94.1	34.2	4.9	61.0	986.5	95	737
TKK_05SA_0052	150400	4485	3.0	1203	233	1945	3381	75.4	35.6	6.9	57.5	491.5	44	419
TKK_05SA_0054	161050	7287	4.5	2728	632	3144	6504	89.3	41.9	9.7	48.3	1060.5	130	658
TKK_05SA_0055	124438	5311	4.3	1049	1041	2530	4620	87.0	22.7	22.5	54.8	469	328	782
TKK_05SA_0010	164507	8186	5.0	4863	697	1380	6940	84.8	70.1	10.0	19.9	2139	277	394

Table S10. Percentage of H37Rv reads mapping to each of the three RPA amplicons.

	% of H37Rv reads mapping to:		
	rpoB	***katG***	***inhA***
**Mean**	37.5	16.3	46.2
**Median**	35.4	15.3	43.7
**Variance**	357.4	75.4	333.9
**Standard deviation**	18.9	8.7	18.3
**Standard error**	3.5	1.6	3.4

Table S11. Drug resistance mutations used, taken from the systematic review by Miotto *et al.*[6]

				Mutation 1					Mutation 2				
No	Gene region	Mutation	Confidence	Codon	Codon begin	Codon end	Wt	Mut	Codon	Codon begin	Codon end	Wt	Mut
1	*rpoB*	F505V+D516Y	High	505	761076	761078	TTC	GTC	516	761109	761111	GAC	TAC
2	*rpoB*	S512T	High	512	761097	761099	AGC	ACC					
3	*rpoB*	Q513H+L533P	High	513	761100	761102	CAA	CAC	533	761124	761126	CTG	CCG
4	*rpoB*	Q513-F514ins	High	513/514	761100	761102	CAA	/					
5	*rpoB*	Q513K	High	513	761100	761102	CAA	AAA					
6	*rpoB*	Q513L	High	513	761100	761102	CAA	CTA					
7	*rpoB*	Q513P	High	513	761100	761102	CAA	CCA					
8	*rpoB*	F514dupl	High	514	761103	761105	TTC	/					
9	*rpoB*	M515I+D516Y	High	515	761106	761108	ATG	ATC	516	761109	761111	GAC	TAC
10	*rpoB*	D516A	High	516	761109	761111	GAC	GCC					
11	*rpoB*	D516F	High	516	761109	761111	GAC	TTC					
12	*rpoB*	D516G	High	516	761109	761111	GAC	GGC					
13	*rpoB*	D516G+L533P	High	516	761109	761111	GAC	GGC	533	761124	761126	CTG	CCG
14	*rpoB*	D516ins	High	516	761109	761111	GAC	/					
15	*rpoB*	D516N	High	516	761109	761111	GAC	AAC					
16	*rpoB*	D516V	High	516	761109	761111	GAC	GTC					
17	*rpoB*	Del N518	High	518	761115	761117	AAC	/					
18	*rpoB*	S522Q	High	522	761127	761129	TCG	CAG					
19	*rpoB*	H526C	High	526	761139	761141	CAC	TGC					
20	*rpoB*	H526D	High	526	761139	761141	CAC	GAC					
21	*rpoB*	H526F	High	526	761139	761141	CAC	TTC					
22	*rpoB*	H526G	High	526	761139	761141	CAC	GGC					
23	*rpoB*	H526L	High	526	761139	761141	CAC	CTC					
24	*rpoB*	H526R	High	526	761139	761141	CAC	CGC					
25	*rpoB*	H526Y	High	526	761139	761141	CAC	TAC					
26	*rpoB*	S531F	High	531	761154	761156	TCG	TTC					
27	*rpoB*	S531L	High	531	761154	761156	TCG	TTG					
28	*rpoB*	S531Q	High	531	761154	761156	TCG	CAG					
29	*rpoB*	S531W	High	531	761154	761156	TCG	TGG					
30	*rpoB*	S531Y	High	531	761154	761156	TCG	TAG					
31	*rpoB*	D626E	High	626	761439	761441	GAC	GAG					
32	*rpoB*	D516Y	Mod	516	761109	761111	GAC	TAC					
33	*rpoB*	S522L	Mod	522	761127	761129	TCG	TTG					
34	*rpoB*	H526P	Mod	526	761139	761141	CAC	CCC					
35	*rpoB*	L533P	Mod	533	761124	761126	CTG	CCG					
36	*rpoB*	L511P	Min	511	761094	761096	CTG	CCG					
37	*rpoB*	H526N	Min	526	761139	761141	CAC	AAC					
38	*rpoB*	I572F	Min	572	761277	761279	ATC	TTC					
39	*katG*	S315I	High	315	2155169	2155167	AGC	ATC					
40	*katG*	S315N	High	315	2155169	2155167	AGC	AAC					
41	*katG*	S315T	High	315	2155169	2155167	AGC	ACC					
42	*katG*	Pooled frameshifts	High	Any									
43	*katG*	Premature stop codons	High	Any									
44	*inhA*	g-102a	High	/			g	a					
45	*inhA*	c-15t	High	/			c	t					

Mod: moderate; Min: minimal; Wt: wild-type; Mt: Mutation

Table S12. Rifampicin resistance mutations detected by nanopore sequencing, Illumina sequencing and the original drug susceptibility testing (DST) result.

Sample	*rpoB* mutations detected by RPA/nanopore sequencing	RPA/Nanopore DR prediction	Mutations detected by Illumina WGS outside of region targeted by RPA	Illumina WGS DR prediction	RIF DST result
TKK_05MA_0004	S531L (High)	R		R	R
TKK_05MA_0009	H526L (High	R		R	R
TKK_05MA_0033	S531L (High)	R		R	R
TKK_05MA_0035	S531L (High)	R		R	R
TKK_05MA_0037	S531L (High)	R		R	R
TKK_05MA_0040	S531L (High)	R		R	R
TKK_05MA_0051	Q490R*, S531W (High), L635*	R		R	R
TKK_05MA_2005	D516G (High), L533P (Mod)	R		R	R
TKK_05MA_2008	D516G (High), L533P (Mod)	R		R	R
TKK_05MA_2015	D516V (High)	R		R	R
TKK_05SA_0010	S531L (High)	R		R	R
TKK_05SA_0011	S531L (High), V661P*	R		R	R
TKK_05SA_0014	L511P (Min), D516Y (Mod)	R		R	R
TKK_05SA_0016	S531L (High)	R		R	R
TKK_05SA_0018	S531L (High)	R		R	R
TKK_05SA_0019	S531L (High)	R		R	R
TKK_05SA_0020	D516V (High)	R		R	R
TKK_05SA_0021	/	S		S	S
TKK_05SA_0024	L511P (Min)	S		S	S
TKK_05SA_0025	S531L (High)	R		R	R
TKK_05SA_0042	S531L (High)	R		R	R
TKK_05SA_0043	D516 (High), L533P (Mod)	R		R	R
TKK_05SA_0044	S531L (High)	R		R	R
TKK_05SA_0046	D516DEL*	S		R	R
TKK_05SA_0048	S531L (High)	R		R	R
TKK_05SA_0050	H526R (High)	R		R	R
TKK_05SA_0052	D516Y (Mod)	R		R	R
TKK_05SA_0054	D516V (High)	R		R	R
TKK_05SA_0055	S531L (High)	R		R	R

*Not listed as High/Moderate/Minimal confidence mutations by Miotto et al.

Table S13. Isoniazid resistance mutations detected by nanopore sequencing, Illumina sequencing and the original drug susceptibility testing (DST) result.

Sample	*katG* mutations detected by RPA/nanopore sequencing	*inhA* mutations detected by RPA/nanopore sequencing	RPA/ Nanopore DR prediction	Mutations detected by Illumina WGS outside of regions targeted by RPA	Illumina WGS DR prediction	INH DST result
TKK_05MA_0004	S315T (High)		R		R	R
TKK_05MA_0009	S315T (High)		R		R	R
TKK_05MA_0033		c(-15)t (High), I21T*	R		R	R
TKK_05MA_0035	S315T (High)		R		R	R
TKK_05MA_0037	S315T (High)		R		R	R
TKK_05MA_0040		c(-15)t (High), I21T*	R		R	R
TKK_05MA_0051	S315T (High)		R		R	R
TKK_05MA_2005	S315T (High)	t(-8)a*	R		R	R
TKK_05MA_2008	S315T (High)	t(-8)a*	R		R	R
TKK_05MA_2015	S315T (High)		R		R	R
TKK_05SA_0010	S315T (High)		R		R	R
TKK_05SA_0011	S315T (High)		R		R	R
TKK_05SA_0014	S315T (High)		R		R	R
TKK_05SA_0016	S315T (High)		R		R	R
TKK_05SA_0018	S315T (High)	c(-15)t (High)	R		R	R
TKK_05SA_0019	R463L*		S	*mshA* A187V	R	S
TKK_05SA_0020	S315T (High)	t(-8)a*	R		R	R
TKK_05SA_0021			S		S	S
TKK_05SA_0024			S		S	S
TKK_05SA_0025	S315T (High)		R		R	R
TKK_05SA_0042	S315T (High)	t(-8)a*	R		R	R
TKK_05SA_0043	S315T (High)	t(-8)a*	R		R	R
TKK_05SA_0044	S315T (High), D448A*		R		R	R
TKK_05SA_0046	R463L*		S	*mshA* A187V	R	S
TKK_05SA_0048	S315T (High), R463*	t(-8)a*	R		R	R
TKK_05SA_0050	S315T (High)		R		R	R
TKK_05SA_0052	S315T (High), R463*		R	*mshA* A187V	R	R
TKK_05SA_0054	S315T (High)		R		R	R
TKK_05SA_0055	S315T (High)		R		R	R

*Not listed as High/Moderate/Minimal confidence mutations by Miotto et al.

Table S14. Two-by-two table for the assessment of diagnostic accuracy of RIF resistance prediction by Illumina WGS, using phenotypic drug susceptibility testing (DST) as the reference test (where R denotes RIF resistant, and S denotes RIF susceptible).

		Reference (DST)				
		**R**	**S**	**Total**	**Sensitivity (95% CI)**	**Specificity (95% CI)**
**Index (Illumina WGS)**	**R**	26	0	26	96.30 (81.03 – 99.91%)	100.00% (15.81 - 100.00%)
	**S**	1	2	3		
	**Total**	27	2	29		

Table S15. Two-by-two table for the assessment of diagnostic accuracy of INH resistance prediction by Illumina WGS, using phenotypic drug susceptibility testing (DST) as the reference test (where R denotes INH resistant, and S denotes INH susceptible).

		Reference (DST)				
		**R**	**S**	**Total**	**Sensitivity (95% CI)**	**Specificity (95% CI)**
**Index (Illumina WGS)**	**R**	25	2	27	100.00% (86.28 – 100.00%)	50.00% (6.76% - 93.24%)
	**S**	0	2	2		
	**Total**	25	4	29		

Table S16. Median read depths for samples selected for "time to prediction" analysis across each amplicon.

**Sample**	**Median read depth**		
	**rpoB**	**katG**	**inhA**
**TKK_05SA_0021**	159	146	386
**TKK_05SA_0016**	865	220	554
**TKK_05MA_2008**	1694.5	370	1055

Table S17. Estimating time to prediction by subsampling reads from three CUBS samples (TKK_05SA_0021, TKK_05SA_0016 and TKK_05MA_2008).

Sample	Total reads used	Proportion of reads used (%)	Sequencing time per sample (seconds)	Number of reads mapping to TB	*rpoB*			*katG*			*inhA*		
					Median depth	Substitution errors	Deletions / missing bases	Median depth	Substitution errors	Deletions / missing bases	Median depth	Substitution errors	Deletions / missing bases
TKK_05SA_0021	51363	50	450	1547	89	0	0	77	0	0	191	0	0
	25682	25	225	805	42	0	0	39	0	0	106	0	0
	10273	10	90	309	20	0	0	13	0	3	42	0	0
	5136	5	45	155	9	0	4	6	1	11	18	0	2
	1027	1	9	42	1	8	292	2	5	86	5	0	27
TKK_05SA_0016	43689	50	450	4142	495	0	0	101	0	0	269	0	0
	21844	25	225	2078	242	0	0	53	0	0	145	0	0
	8738	10	90	878	105	0	0	23	0	0	51	0	0
	4369	5	45	445	50	0	0	14	0	2	25	0	0
	874	1	9	82	8	0	3	2	2	139	7	0	4
TKK_05MA_2008	97512	50	450	5963	932	0	0	190	0	0	549	0	0
	48756	25	225	2977	487	0	0	92	0	0	248	0	0
	19502	10	90	1198	194	0	0	37	0	0	108	0	0
	9751	5	45	587	93	0	0	20	0	0	54	0	0
	1950	1	9	122	19	0	0	4	1	21	12	0	2

Table S18. Costs of the RPA/nanopore workflow, including DNA extraction using the MolYsis DNA extraction kit, as used by Votintseva et al.[3]

**Component**	**Cost per sample (assume 12 barcoded samples per flow cell) (GBP)**
Molysis DNA extraction	9.70
RPA reagents	2.50
FFPE DNA repair mix & buffer	5.20
Ultra II End prep mix & buffer	7.20
Blunt/TA Ligase Master Mix	7.60
Quick ligation module	2.15
Ligation Sequencing kit	6.66
Native barcoding kit	3.26
Flow cell	52.92
Agencourt beads	1.20
TOTAL	98.39

References

1. Warren AS, Setubal JC. The Genome Reverse Compiler: an explorative annotation tool. *BMC Bioinformatics* 2009; **10**: 35.
2. Camus JC, Pryor MJ, Medigue C, Cole ST. Re-annotation of the genome sequence of Mycobacterium tuberculosis H37Rv. *Microbiology* 2002; **148**(Pt 10): 2967-73.
3. Ye J, Coulouris G, Zaretskaya I, Cutcutache I, Rozen S, Madden TL. Primer-BLAST: A tool to design target-specific primers for polymerase chain reaction. *Bmc Bioinformatics* 2012; **13**.
4. Schneider CA, Rasband WS, Eliceiri KW. NIH Image to ImageJ: 25 years of image analysis. *Nat Methods* 2012; **9**(7): 671-5.
5. Cohen KA, Abeel T, Manson McGuire A, et al. Evolution of Extensively Drug-Resistant Tuberculosis over Four Decades: Whole Genome Sequencing and Dating Analysis of Mycobacterium tuberculosis Isolates from KwaZulu-Natal. *PLoS Med* 2015; **12**(9): e1001880.
6. Miotto P, Tessema B, Tagliani E, et al. A standardised method for interpreting the association between mutations and phenotypic drug resistance in *Mycobacterium tuberculosis*. *European Respiratory Journal* 2017; **50**(6).
7. Larsen MH, Biermann K, Tandberg S, Hsu T, Jacobs WR, Jr. Genetic Manipulation of Mycobacterium tuberculosis. *Curr Protoc Microbiol* 2007; **Chapter 10**: Unit 10A 2.
8. Sandgren A, Strong M, Muthukrishnan P, Weiner BK, Church GM, Murray MB. Tuberculosis drug resistance mutation database. *PLoS Med* 2009; **6**(2): e2.
9. Joshi KR, Dhiman H, Scaria V. tbvar: A comprehensive genome variation resource for Mycobacterium tuberculosis. *Database (Oxford)* 2014; **2014**: bat083.
10. Coll F, Preston M, Guerra-Assuncao JA, et al. PolyTB: a genomic variation map for Mycobacterium tuberculosis. *Tuberculosis (Edinb)* 2014; **94**(3): 346-54.
11. Chernyaeva EN, Shulgina MV, Rotkevich MS, et al. Genome-wide Mycobacterium tuberculosis variation (GMTV) database: a new tool for integrating sequence variations and epidemiology. *BMC Genomics* 2014; **15**: 308.

3\. Votintseva AA, Bradley P, Pankhurst L, et al. Same-Day Diagnostic and Surveillance Data for Tuberculosis via Whole-Genome Sequencing of Direct Respiratory Samples. *J Clin Microbiol* 2017; 55(5): 1285-98.
